# Modulation of Autophagy by Sorafenib: Effects on Treatment Response

**DOI:** 10.3389/fphar.2016.00151

**Published:** 2016-06-08

**Authors:** Nestor Prieto-Domínguez, Raquel Ordóñez, Anna Fernández, Andres García-Palomo, Jordi Muntané, Javier González-Gallego, José L. Mauriz

**Affiliations:** ^1^Centro de Investigación Biomédica en Red de Enfermedades Hepáticas y Digestivas (CIBERehd)León, Spain; ^2^Institute of Biomedicine (IBIOMED), University of LeónLeón, Spain; ^3^Service of Clinical Oncology, Complejo Asistencial Universitario de León (Hospital of León)León, Spain; ^4^Department of General Surgery“Virgen del Rocío”-“Virgen Macarena” University Hospital/IBiS/CSIC/Universidad de Sevilla, Spain

**Keywords:** sorafenib, autophagy, hepatocellular carcinoma, cancer therapeutic, dug resistance

## Abstract

The multikinase inhibitor sorafenib is, at present, the only drug approved for the treatment of hepatocellular carcinoma (HCC), one of the most lethal types of cancer worldwide. However, the increase in the number of sorafenib tumor resistant cells reduces efficiency. A better knowledge of the intracellular mechanism of the drug leading to reduced cell survival could help to improve the benefits of sorafenib therapy. Autophagy is a bulk cellular degradation process activated in a broad range of stress situations, which allows cells to degrade misfolded proteins or dysfunctional organelles. This cellular route can induce survival or death, depending on cell status and media signals. Sorafenib, alone or in combination with other drugs is able to induce autophagy, but cell response to the drug depends on the complex integrative crosstalk of different intracellular signals. In cancerous cells, autophagy can be regulated by different cellular pathways (Akt-related mammalian target of rapamycin (mTOR) inhibition, 5′ AMP-activated protein kinase (AMPK) induction, dissociation of B-cell lymphoma 2 (Bcl-2) family proteins from Beclin-1), or effects of some miRNAs. Inhibition of mTOR signaling by sorafenib and diminished interaction between Beclin-1 and myeloid cell leukemia 1 (Mcl-1) have been related to induction of autophagy in HCC. Furthermore, changes in some miRNAs, such as miR-30α, are able to modulate autophagy and modify sensitivity in sorafenib-resistant cells. However, although AMPK phosphorylation by sorafenib seems to play a role in the antiproliferative action of the drug, it does not relate with modulation of autophagy. In this review, we present an updated overview of the effects of sorafenib on autophagy and its related activation pathways, analyzing in detail the involvement of autophagy on sorafenib sensitivity and resistance.

## Introduction

Hepatocellular carcinoma (HCC) is the most common type of liver cancer and the second most frequent cause of cancer-related death worldwide (Ferlay et al., 2015; Torre et al., 2015). The staging and recommended treatment of patients with HCC is related to the liver function, size and number of nodules, general status of the patient, vascular invasion, and the presence of extrahepatic metastasis. Curative treatments such as surgical resection, liver transplantation, and radiofrequency ablation may be useful in the early stages of the disease. However, only palliative treatments are available in advanced stages, in which different chemotherapeutics have been assayed with variable effectiveness (Rossi et al., 2010; Forner et al., 2012). The development of new diagnostic methods which can detect small liver tumors is essential to allow more aggressive interventions and to improve patient survival rates (Gonzalez, 2014).

Sorafenib (BAY 43-9006, Nexavar®), which was developed in 1995 (Gauthier and Ho, 2013), is the only chemotherapeutic drug which has demonstrated to improve survival rate in patients with HCC (Llovet et al., 2008; Abdel-Rahman and Fouad, 2014). Recent studies have also proven that sorafenib has therapeutic effects in other cancer types, such as thyroid cancer, acute myeloid leukemia, advanced renal cell carcinoma or prostate cancer (Escudier et al., 2007; Gollob et al., 2007; Chi et al., 2008; Antar et al., 2014; Luo et al., 2014; Alonso-Gordoa et al., 2015; Yamamoto et al., 2015). Sorafenib targets the RAF serine/threonine kinases, a family of three members (A-RAF, B-RAF, and C-RAF/Raf-1) that play a key role in the transduction of mitogenic and oncogenic signals through the Raf/Mitogen-activated protein (MAP)/extracellular signal-regulated kinase (ERK) kinase (MEK)/ERK signaling pathway, resulting in a lower cyclin D1 expression and in cell cycle arrest (Wellbrock et al., 2004; Adnane et al., 2006; Liu et al., 2006). Sorafenib also potently inhibits tyrosine kinase receptors such as vascular endothelial growth factor receptor (VEGFR) 2, VEGFR 3, platelet-derived growth factor receptor-β (PDGFR-β), Flt3, and c-Kit, which promote angiogenesis (Wilhelm et al., 2004, 2008; Cervello et al., 2012). The repression blocks a broad spectrum of different processes involved in proliferation, angiogenesis or apoptosis, causing a reduction in blood vessel area in the tumor and starving cancerous cells (Erber et al., 2004; Gauthier and Ho, 2013). Furthermore, sorafenib enhances TRAIL-induced cell death through SH2 domain-containing tyrosine phosphatase (SHP-1)-dependent reduction of signal transducers and activators of transcription type 3 (STAT3) phosphorylation (^Tyr705^STAT3) and related proteins Mcl-1 (myeloid cell leukemia 1), survivin, and cyclin D1 in hepatoma cells (Chen et al., 2010). Sorafenib is also able to repress Mcl-1 activity through a MAPK-independent mechanism, which increases the apoptosis intrinsic pathway in tumor cells (Yu et al., 2005; Ulivi et al., 2009). Moreover, recent studies have claimed that eIF4E (eukaryotic translation initiation factor 4E) might be implicated in sorafenib-dependent Mcl-1 inhibition (Rahmani et al., 2005).

Nowadays, sorafenib is the only Food and Drug Administration (FDA)-approved HCC systemic therapy, expanding patient mean survival from 7.9 to 10.7 months (Llovet et al., 2008; Guan and He, 2011). Despite initial response, most patients develop disease progression probably as a consequence of tumor resistant cells which do not respond to this molecule, mainly due to upregulation of some survival pathways which may cover up the death signals induced by sorafenib. Therefore, a better knowledge of those cellular routes is required to overcome unwanted tumor resistance and consequently improve the beneficial effects of sorafenib therapy (Knievel et al., 2014; Sakai et al., 2015; Togashi and Nishio, 2015).

Macroautophagy (hereafter referred to as autophagy) is a bulk degradation system which recycles unfolded, damaged, or useless cellular components, like proteins or organelles, for the maintenance of cellular homeostasis in order to promote adaptation and cell survival (San-Miguel et al., 2014, 2015; Vallejo et al., 2014). However, excessively stimulation may lead to programmed cell death instead of survival (Tsujimoto and Shimizu, 2005). In fact, its deregulation has been associated with some diseases such as neurodegenerative disorders, diabetes, cystic fibrosis, Crohn's disease, diverse myopathies, hepatitis, α-1 trypsin deficiency, cardiac hypertrophy, and tumorigenesis (Mizushima and Komatsu, 2011). Autophagy is initiated with the formation of a small membranous vesicle named phagophore that elongates and engulfs a specific or unspecific portion of the cytoplasm, forming a doubled-membranous structure named autophagosome. Afterwards, this organelle fuses with a lysosome and forms a combined vesicle or autophagolysosome, leading to the degradation of the inner material by lysosomal enzymes, releasing substances that become at disposal for the synthesis of newly macromolecules formation or energy production (Tanida, 2011).

Autophagic process involves a highly conserved group of macromolecules which were discovered in yeasts and named as autophagy-related genes (Atg) (Tanida, 2011). In mammals, five groups of proteins are involve in autophagosome formation (Tanida, 2011). The first is ULK1 (unc-51-like kinase) complex, whose functions are the recruitment of distinct Atg components and the maintenance of phagophore integrity (Mizushima, 2010). Next, Beclin1-Vps34 (vacuolar protein sorting 34) complex allows membrane nucleation with formation of PI3P (phosphatidylinositol 3-phosphate) (Yuan et al., 2013). Afterwards, Atg9 and WIPI-1 (WD-repeat protein interacting with phosphoinositides) system brings to phagophore some lipids and proteins which are necessary for its elongation (Orsi et al., 2010). Finally, two ubiquitin-like systems, Atg5-Atg12-Atg16L and LC3 (microtubule-associated protein 1 light chain 3), that conjugating with PE (phosphatidylethanolamine) constitutes LC3-II (van der Veen and Ploegh, 2012), are both required for autophagosome formation. The first system plays an important role in the activation of the second one, and LC3 protein is implicated in the elongation of autophagosome membrane and in its closure (Sou et al., 2008).

There are various extracellular stimuli, such as nutrient deprivation, growth factor withdrawal or hypoxia, which have the ability to induce autophagy (He and Klionsky, 2009). Stress situations, such as reactive oxygen species (ROS) accumulation or endoplasmic reticulum (ER) stress can also modify this cellular process (He and Klionsky, 2009). The most important pathways involved in autophagy modulation are mTORC1 (mammalian target of rapamycin complex 1) and AMPK (5′ AMP-activated protein kinase). The first one abolishes autophagy in presence of nutrients or growth factors, through ULK1 complex inhibition, while the second one promotes autophagy when cellular energetic status is low (Sengupta et al., 2010; Manwani and McCullough, 2013). On the other hand, some sphingolipids are also able to trigger autophagy (Li et al., 2014; Ordoñez et al., 2015). Specifically, ceramide induces autophagy-associated cell death, whereas sphingosine-1-phosphate induces autophagy-associated survival (Li et al., 2014). The interaction between Beclin-1 and Bcl-2 protein family, as well as the post-transcriptional regulation by miRNAs have been related to regulation of autophagy (Zhou et al., 2011; Sui et al., 2015).

Autophagy acts as a double-edged sword in cancer cells because removes newly mutated cells and damaged mitochondria in the early stage of the disease, but induces survival in hypoxia and ischemia conditions, as well as promotes resistance against some chemotherapeutic drugs and tumor progression at the later phases (Eskelinen, 2011; Choi, 2012). In animal models of hepatocarcinogenesis it has been described that autophagy could play a protecting role during dysplastic phase in normal hepatocytes, but promotes tumor cells growth during the tumor-forming stage (Sun et al., 2013). Those apparently controversial results can be related with the ability of autophagy to reduce oxidative stress and maintain healthy mitochondria preventing the initiation of hepatocarcinogenesis, while it blocks the expression of p53 and other tumor suppressors during late phases to promote the development of HCC (Tian et al., 2015). Furthermore, some drugs used in cancer treatment induce autophagy-related cell death in cancer cells (Scarlatti et al., 2004). Thus, it is interesting to review the effects of this antitumor agent on the autophagy process in HCC cells, and the influence of autophagy on sorafenib-related cell resistance generation (Liu et al., 2016). A better knowledge of the sorafenib autophagy-related mechanisms could contribute to improve its therapeutic efficiency, increasing cancer patient survival rates.

## Autophagy induction by sorafenib

Autophagy is usually deregulated in tumor cells whereas has been related to cell survival and drug resistance. Sorafenib and other chemotherapeutic drugs have been shown to modulate autophagy in different *in vitro* and *in vivo* experimental models (Gauthier and Ho, 2013). Sorafenib regulates autophagy in various hepatocellular cell lines (Table 1). In particular, different studies have shown the presence of acid vesicles which are typical features of autophagosomes in sorafenib-treated cells (Table 1; Park et al., 2008; Chiou et al., 2010; Shi et al., 2011; Shimizu et al., 2012; Eum et al., 2013; Honma and Harada, 2013; Tai et al., 2013; Fischer et al., 2014; Stiuso et al., 2015). Moreover, sorafenib can also promote LC3 lipidation, an obvious sign of autophagy induction (Tai et al., 2013; Yuan et al., 2014; Zhai et al., 2014). For example, it has been observed that LC3-II formation by sorafenib is dose-dependent and time-dependent in HepG2, MHCC97-L, Huh7, HLF, and PLC/PRF/5 HCC cells (Shi et al., 2011; Shimizu et al., 2012). In addition, sorafenib modulates the expression of multiple autophagy markers. Thus, the drug stimulates Beclin1, Atg5, and Atg12 expression in HCC cells *in vitro* (Yuan et al., 2014), Beclin-1 expression is increased by sorafenib in a time-dependent fashion in Hep3B cells (Carr et al., 2013), sorafenib can mildly induce Beclin1 and Atg-5 expression whereas decreases p62 expression in a significant manner in PLC-5 cells (Tai et al., 2013), or increases Atg5, Vps34 and Beclin-1, decreases p62 and does not affect UVRAG expression in Huh7 and HepG2 cells (Zhai et al., 2014). Moreover, autophagy induced by sorafenib reaches the lysosome degradatory phase, as demonstrated by using a mRFP-GFP-LC3 combined fluorescent-tag (Shimizu et al., 2012). Several discrepancies in autophagy induction have been also observed in sorafenib-treated HCC cells (Chiou et al., 2010; Fischer et al., 2014). This apparently paradoxical discrepancy and others, about the effects of sorafenib in the autophagic mechanisms, are included in the Table 2. Sorafenib also regulated autophagy in non HCC cancer cells (Table 3) such as macrophages, osteosarcoma, multiple myeloma, colorectal carcinoma cells, prostate, mammary, thyroid, and renal cancer cells, (Walker et al., 2009; Ullen et al., 2010; Bareford et al., 2011a,b; Pfisterer et al., 2011; Kharaziha et al., 2012, 2013; Lin et al., 2013; Zheng et al., 2015) through different pathways which are summarized in the Table 3.

**Table 1:** **Effect of sorafenib on autophagy markers in HCC ***in vitro*** or *in vivo* models**.

**References**	**Model**	**Effects on autophagy markers**	**Global effects**	**Role of autophagy**
Carr et al., 2013	Regorafenib in Hep3B, PLC/PRF/5, HepG2	↑LC3-II ↑Beclin-1	Increase apoptosis on tumor cells (it increases caspase 3, 8, and 9 activity, Bax expression, and decreases Bcl-2 protein levels)	Not assessed
Eum et al., 2013	Multidrug-resistant Ras-NIH 3T3/Mdr cells	↑LC3-positive vesicles	Sorafenib raises HCC cell death through the activation of autophagy pathway and the inhibition of mTOR activity	Cell death
Fischer et al., 2014	Hep3B, HuH7	↑LC3 lipidation only in HuH7, but with no effects in Hep3B cells	Those cell lines have different autophagy responsiveness to sorafenib and that might be linked to generation of sorafenib resistant cells	Not assessed
Shi et al., 2011	MHCC97-L, PLC/PRF/5, HepG2	↑Autophagosome formation ↑LC3 lipidation ↑Atg5	IRE-1α signaling pathway of ER stress is necessary for autophagy induction by sorafenib	Cell survival
Shi et al., 2011	Nude mice ortothopically implanted with MHCC97-L	↑CHOP	Autophagy inhibition decrease tumor volume in sorafenib and sorafenib + cloroquine treated mice	Cell survival
Shimizu et al., 2012	HuH7, HLF, PLC/PRF/5	↑LC3 lipidation ↓p62 ↑Autophagosome formation = Atg5 and Beclin-1	Inhibition of autophagy increases cell sensitivity to sorafenib	Cell survival
Shimizu et al., 2012	Xenograft tumors of HuH7 cells in nude mice	↑LC3 lipidation	Autophagy inhibition decrease tumor volume in Sorafenib treated mice	Cell survival
Tai et al., 2013	PLC/PRF/5, Hep3B, Sk-Hep-1, HepG2	↑LC3 lipidation ↓p62 ↑Beclin-1 and Atg5	Sorafenib induces autophagy through the disruption of Beclin-1-Mcl-1 complex	Cell death
Tai et al., 2013	Nude mice injected with PLC-5	Autophagy induction	Sorafenib induces autophagy *in vivo* through p-STAT inhibition and abolishes cancer proliferation	Cell death
Zhai et al., 2014	Sorafenib-resistant and sensitive HepG2 and HuH7 cells.	↑LC3-II. Atg5, Vps34, Beclin1 ↓p62 in both, resistant and sensitive cells although resistant cells show lower levels of that markers	Lack of sorafenib-induced autophagy in HCC cells leads to generation of sorafenib-resistant cells	Protection in parental cells, but promotion of cell death in resistant cell lines
Zhai et al., 2014	Mice injected with HuH7 resistant cells	Results in accordance to *in vitro* assay	Autophagy behavior switch is able to modify cell sensitiveness to sorafenib.	Results in accordance to *in vitro* assay

**Table 2:** **Discrepancies existent between different sorafenib either ***in vivo*** or ***in vitro*** studies**.

**Condition**	**First situation**	**Second situation**
LC3	↑Levels in Huh7 cells, inducing more drug response (Fischer et al., 2014)	Moderate ↑ levels in Hep3B cells, inducing less drug response (Fischer et al., 2014).
Autophagy	↑Levels, protecting cancerous cells from cell death both *in vitro* an *in vivo* models (Shimizu et al., 2012)	↑Levels, promoting programmed cellular death in cancerous cells both *in vitro* an *in vivo* models (Tai et al., 2013)
Autophagy	↓Levels in drug combination, reducing side effects of both drugs (Manov et al., 2011)	↑ Levels in drug combination, leading to enhance drug synergism (Yuan et al., 2014)
mTORC1	↓Phosphorylation, leading to autophagy induction and cell death (Zhai et al., 2014; Zhang et al., 2015)	No changes in phosphorylation associated with sorafenib cell resistance (Ramakrishnan et al., 2012)
mTORC1	↓Phosphorylation by some of the analogs of sorafenib associated with autophagy (Tavallai et al., 2015)	No changes in phosphorylation by some of the analogs of sorafenib, not being autophagy induction dependent on that pathway (Wecksler et al., 2014)
Akt	↓Expression associated with increments in cellular death both *in vivo* and *in vitro* experiments (Eulitt et al., 2010)	↑Levels both *in vivo* and *in vitro* models, leading to induction of survival autophagy, survival pathways, and increasing sorafenib cell resistance (Zhai et al., 2014)
AMPK	↑Phosphorylation due to the generation of a reduction of ATP cellular levels (Tesori et al., 2015)	↑Phosphorylation due to the release of ROS from mitochondria (Pignochino et al., 2015)
AMPK	↑Phosphorylation without autophagy associated (Tesori et al., 2015)	No changes in AMPK activation and no autophagy associated (Sviripa et al., 2013)
ER stress	↑IRE-1α expression, leading to autophagy induction (Shi et al., 2011)	↑PERK expression, but non-autophagy induction associated (Shi et al., 2011)
Sphingolipids	↑Ceramide formation at *in vitro* models, leading to induction of autophagy and programmed cell death (Park et al., 2010)	↓Slightly S1P levels at *in vivo* models (Beljanski et al., 2011)

**Table 3:** **Effect of sorafenib on autophagy markers in other ***in vitro*** or ***in vivo*** cancerous models distinct of HCC**.

**References**	**Model**	**Effects on autophagy markers**	**Global effects**	**Role of autophagy**
Lin et al., 2013	Human macrophages	↑Autophagic vacuoles ↑LC3 lipidation	Sorafenib stimulates autophagy but it inhibits phagocytosis and secretion of IL-10	Not assessed
Kharaziha et al., 2012	Myeloma cell lines LP1, RPMI-8226	↑LC3 lipidation ↓p62 ↑LC3-positive vesicles	Sorafenib induces autophagy which protect against caspase-dependent and independent cell death	Cell survival
Kharaziha et al., 2012	Myeloma patient samples	↑LC3 lipidation	It induces cell death and autophagy	Cell death
Kharaziha et al., 2012	Myeloma mice models	↑LC3 lipidation	Sorafenib increases mice survival, reduces tumor development and induces autophagy pathway	Cell death
Walker et al., 2009	Sorafenib plus vorinostat treatment in a colorectal cancer cell line, HCT116	↑LC3 lipidation ↑Atg5	Sorafenib treatment induces a slightly amount of autophagy which is cytoprotective and is stimulated by vorinostat cotreatment	Cell survival
Ullen et al., 2010	Prostate carcinoma cell lines DU145 and PC3	↑Autophagosome formation	Sorafenib alters mitochondrial potential and induces apoptosis and autophagy	Not assessed
Bareford et al., 2011b	Fulvestrant-resistant and sensitive MCF7 cell line (originating from breast adenocarcinoma)	↑Autophagosome formation, ↑Beclin-1 ↑Atg5-Atg12 ↑LC3-II ↓p62	Sorafenib induces autophagy pathway alone or in combination with permetrexed, and that induction sensitizes that cells to cell death	Cell death
Pfisterer et al., 2011	Human osteosarcoma cell line U2OS	↑WIPI ↑Autophagosome formation	Sorafenib induces autophagy in normal and starved cells and that is induced by calcium ion release to cytoplasm	Not assessed
Lin et al., 2012	Medullary thyroid cancer cell lines MTG-1 and TT	↑LC3-II↑Atg5	Sorafenib induces both autophagy and apoptosis in that *in vitro* model	Cell death
Zheng et al., 2015	Diverse renal carcinoma cell lines like 786-0, A498 and SK-RC-44	↑Beclin-1 ↑Atg5 ↓p62 ↑LC3-II and LC3-II/I ratio	Sorafenib induces a cytoprotective form of autophagy in renal carcinoma cells	Cell survival

Some studies connect sorafenib administration and autophagy modulation in experimental HCC models *in vivo* (Tables 1, 3). In all research done until now, sorafenib can increase autophagosome formation and modify autophagy markers expression in a similar way to models *in vitro* (Shi et al., 2011; Shimizu et al., 2012; Tai et al., 2013). The only study analyzing the potential link between sorafenib and autophagy in patients has been performed in refractory or relapsed lymphoproliferative disease, demonstrating that LC3-II base levels are lower in non-responsible patients compared to responders, and that patients who respond to sorafenib show a higher reduction of LC3 expression after 1 month of treatment (Guidetti et al., 2012).

Regorafenib, a structural analog of sorafenib, induces autophagosome formation in HCC cells similarly than sorafenib (Carr et al., 2013; Tavallai et al., 2015). In another study, two different sorafenib analogs, t-MTUCB and AUCMB, caused autophagosome formation and LC3 lipidation in various HCC cell lines (Wecksler et al., 2014). Finally, sc-59, a kinase-independent derivate of sorafenib showed a higher autophagy induction characterized by an increased ability to induce LC3-lipidation and acid vesicles formation (Tai et al., 2013).

Some works have established that sorafenib induces autophagy as a cellular survival mechanism in HCC because when this pathway is repressed by a chemical drug (like chloroquine or bafilomycin A1) or by a small interfering RNA (siRNA) against Beclin-1 or Atg5, sorafenib kills more cancerous cells and its antiproliferative ability improves, which means that autophagy induced by that antitumor agent works as a protective pathway (Park et al., 2010; Yuan et al., 2014). Moreover, similar effects have been also described in other non HCC cancer cells (Tables 1, 3; Martin et al., 2009; Walker et al., 2009; Kharaziha et al., 2012; Zheng et al., 2015). However, other studies have observed opposite results in HCC (Tables 1, 3; Eum et al., 2013; Tai et al., 2013; Tavallai et al., 2015) and non-HCC cells (Lian et al., 2012; Lin et al., 2012). Tumor cell resistance to sorafenib can also be related to switch from autophagy-related death to autophagy-related HCC cell survival (Zhai et al., 2014). Moreover, another different way to generate sorafenib resistance consists in the abolishment of autophagy induction. In fact, it has been demonstrated that sorafenib resistant cells show lower levels of autophagy markers such as LC3, Atg5, Vps34, or Beclin1 (Zhai et al., 2014). For all these reasons, normalization of autophagy may be one of the key mechanisms to avoid cellular resistance to that antineoplastic agent (Zhai et al., 2014; Liu et al., 2016).

## Effects on autophagy of sorafenib combination with other drugs

Some studies have analyzed changes in autophagy modulation in HCC cells treated with different sorafenib-based drug combinations (Table 4). The administration of an inhibitor of histone deacetylases (HDAC), enzyme implicated in the regulation of gene transcription related to promotion or progression of cancer (Dokmanovic et al., 2007; Giannini et al., 2012), has been shown to suppress tumor cell proliferation. The most important member of that group is vorinostat, the first HDACi to be approved for human clinical use (Giannini et al., 2012). When vorinostat is combined with sorafenib in HCC cultured cells, cell viability decreases more than in cells treated with sorafenib or, vorinostat alone (Park et al., 2008, 2010). Furthermore, drug combination can induce a higher Beclin-1, Atg5, or Atg12 expression and LC3 lipidation than treatment with only one chemotherapeutic agent (Park et al., 2008; Yuan et al., 2014). When Atg5 or Beclin-1 were silenced with siRNA in those studies, viability decreased more in cancer cells than in control cells, being the drop higher in the drug combination treated group (Park et al., 2008; Yuan et al., 2014). This result is compatible with the prosurvival properties of autophagy during the antiproliferative synergistic effect of those drugs (Table 4; Park et al., 2008; Martin et al., 2009; Yuan et al., 2014).

**Table 4:** **Effect of sorafenib combined treatment with another drug on autophagy in HCC and other cancer types**.

**Reference**	**Pathophysiological condition**	**Model**	**Drug combined with sorafenib**	**Sorafenib alone effects**	**Drug combination effects**	**Global effect of both drugs treatment**
Yuan et al., 2014	Hepatocellular carcinoma	*In vitro*	Vorinostat	↑Beclin-1 ↑Atg5 ↑Atg7 ↑LC3-II	↑↑Beclin-1 ↑↑Atg5 ↑↑Atg7 ↑↑LC3-II	Drug combination enhances Beclin-1-dependant protective form of autophagy
Manov et al., 2011	Hepatocellular carcinoma	*In vitro*	Doxorubicin	↑LC3-II	↓LC3-II	Sorafenib acts as an antagonist of doxorubicin
Tavallai et al., 2015	Hepatocellular carcinoma	*In vitro*	Sildenafil	↑Autophagic vacuoles = LC3 = p62	↑↑Autophagic vacuoles ↑LC3 ↓p62	Sidenafil promotes sorafenib effects on autophagy and stimulate autophagic cell death
Lam et al., 2015	Hepatocellular carcinoma	*In vivo*	PHY906	↑LC3 ↓ULK1	↑↑LC3 ↑ULK1	PHY906 stimulate sorafenib-related autophagy
Bareford et al., 2011b	Breast adenocarcinoma	*In vitro*	Pemetrexed	↑Beclin-1 ↑Atg5-Atg12 ↑LC3-II ↓p62	↑↑Beclin-1 ↑↑Atg5-Atg12 ↓slightly LC3-II ↓p62	Permetrexed promotes sorafenib-related autophagy induction
Hamed et al., 2015	Glioblastoma	*In vitro*	Lapatinib	↑Autophagic vacuoles = p62 ↑Beclin-1 ↑LC3-II/I ↓LAMP2	↑↑autophagic vacuoles ↓p62 = Beclin-1 ↓LC3-II/I ↓LAMP2	Lapatinib promotes sorafenib cellular death through autophagy pathway
Jakubowicz-Gil et al., 2014	Glioblastoma multiforme and anaplastic astrocytoma	*In vitro*	Quercetin	↑Autophagic vesicles, Beclin-1 and LC3-I/II only in glioblastomamultiforme cell line.	↑↑Autophagic vesicles, Beclin-1 and LC3-I/II only in the same cell line as sorafenib alone	Quercetin promotes sorafenib cell death in both cell lines but only induces autophagy in one of them
Lian et al., 2012	Androgen-independent prostate cancer	*In vitro* and *in vivo*	(–) gossypol	↑Autophagic vesicles ↑LC3-I/II ↓p62	↑↑Autophagic vesicles ↑↑LC3-I/II ↓↓p62 Preferentially in one of the cell lines tested	(–) gossypol agonists sorafenib autophagy and induces cellular death

The low efficacy of doxorubicin, as a single agent in HCC, has led to evaluate its activity in combined treatment with sorafenib (Manov et al., 2011). Doxorubicin belongs to anthracyclines group, a type of antibiotic which were first isolated from soil bacteria (Yang et al., 2014), that can generate simultaneously topoisomerase II poisoning, DNA adduct formation, ceramide overproduction and oxidative stress (Yang et al., 2014). One of the main problems of the use of doxorubicin alone in therapy is the high number of side effects (Yang et al., 2014). Interestingly, sorafenib decreases doxorubicin-related autophagy, with a reduction of the expression of LC3 and its lipidation (Table 4; Manov et al., 2011).

Sildenafil is another agent which has been combined with sorafenib in HCC (Table 4; Tavallai et al., 2015). This drug is able to inhibit phosphodiesterase 5, an enzyme which transforms cyclic GMP (cGMP) into his inactive form (Das et al., 2015). Sildenafil is used mainly in the treatment of erectile dysfunction and of some cardiovascular diseases because it produces vasodilatation (Das et al., 2015). It has also been postulated that sildenafil is able to induce the intrinsic pathway of apoptosis in colorectal carcinoma cells and lymphatic leukemia cells (Booth et al., 2014; Das et al., 2015). Sildenafil increases the antiproliferative properties of sorafenib and regorafenib (Table 4; Tavallai et al., 2015). The combined treatment increases autophagosome formation followed by the accumulation of red fluorescence at 24 h into GFP-RFP-LC3- transfected HCC cells. Moreover, the reduction of cell death by regorafenib and sildenafil in cells treated with Atg5, Beclin-1, or ULK1 siRNA suggests that autophagy may act as a pro-death mechanism in this setting (Tavallai et al., 2015).

Sorafenib has also been combined with PHY906, an herbal mixture which consists of four distinct components: *Glycyrrhiza uralensis, Paeonia lactiflora, Scutellaria baicalensis* roots, and *Ziziphus jujuba* fruit. This plant mixture is based on an old Chinese formulation used for the treatment of various gastrointestinal diseases, like diarrhea, fever, or vomiting (Liu and Cheng, 2012; Rockwell et al., 2013). Recent studies have shown the efficacy of PHY906 as a chemotherapeutical adjuvant (Kummar et al., 2011). When this herbal mixture is combined with sorafenib, expression of autophagy markers increases. *Paeonia lactiflora* and *Scutellaria baicalensis* are the critical components of that mixture in relation to autophagy induction, because following suppression of those plants from the herbal blend, LC3 is not lipidated (Table 4; Lam et al., 2015).

Similarly, different combinations of sorafenib with other drugs and antioxidants or herbal mixtures seem to be able to modulate autophagy in non-liver cancer cells (see Table 4).

## Autophagy-related cellular pathways and sorafenib treatment

**Figure 1:**
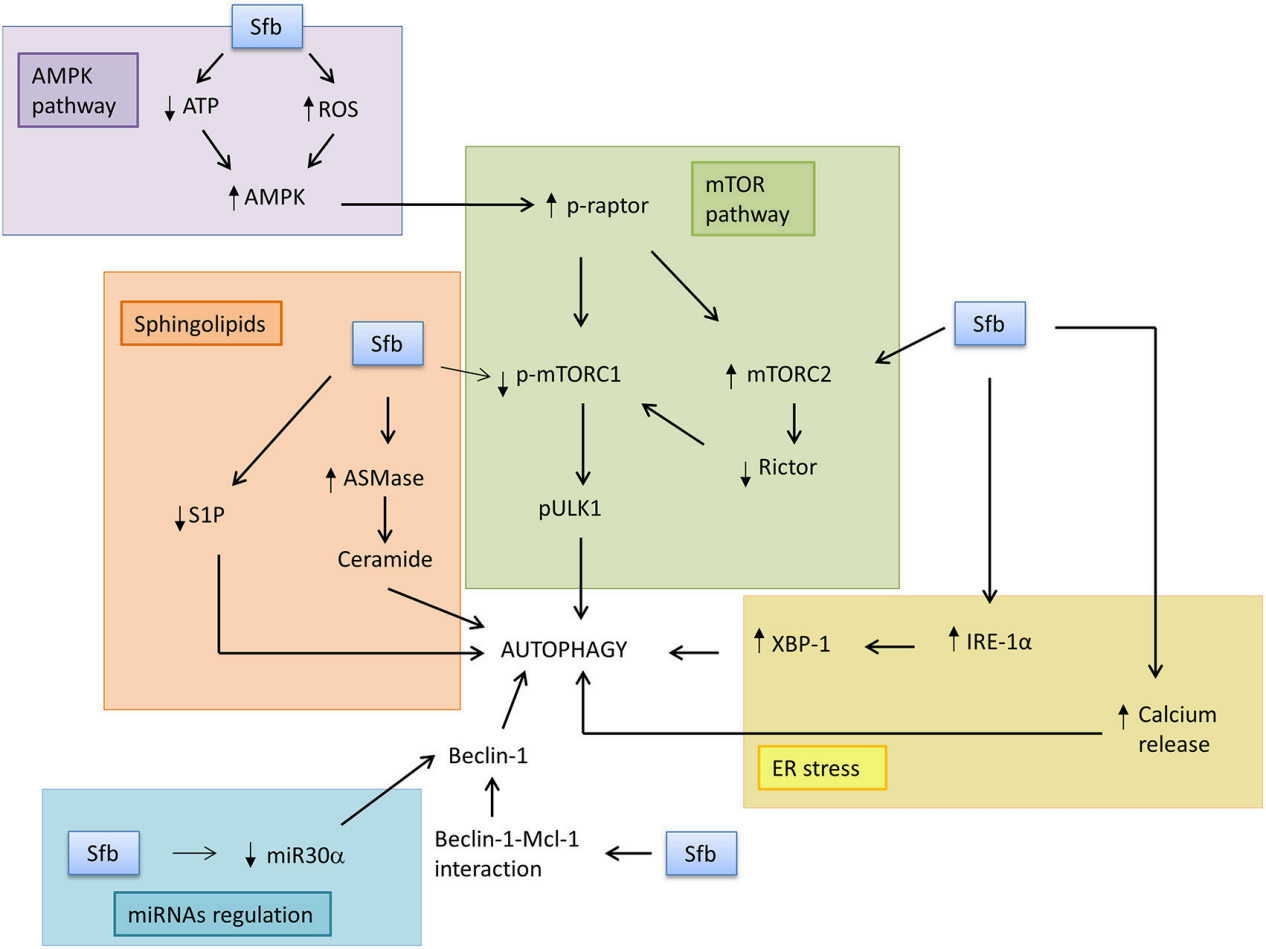
**Sorafenib induces autophagy response through modulation of the main downstream factors and pathways**. In this scheme, some of the mediators of autophagy induction by sorafenib in tumor cells are represented. Sorafenib may induce AMPK pathway because it reduces ATP levels and increases ROS, which leads to inhibition of mTORC1 signaling pathway. Sorafenib can also stimulate ER stress, specifically IRE-1α branch and all its downstream genes, and release calcium ion to cytosol, which induces autophagosome formation. It may also disrupt Beclin-1 and Bcl-2 complex, with Beclin-1 release. Other non-protein mediators which may be involved in sorafenib effects are miRNA30α and sphingolipids, because the drug can reduce miRNA30α signaling, which is a Beclin-1 repressor, induce ceramide formation, and reduce S1P levels, leading to autophagosome formation.

There are some proteins and cellular pathways in tumor cells that can be involved in the regulation of autophagy by sorafenib. Those regulatory routes have a changing effect, because they are dependent on the cell state, its origin or some medium features. The most important are described in detail in the following sections (Figure 1).

### mTOR pathway

The mTOR pathway is one of the main regulators of cellular metabolism in response to oxidative stress, unfolded protein response, hypoxia, nutrients deprivation or growth factor deficiency (Neufeld, 2010; Yang and Ming, 2012; Sarkar, 2013). The inhibition of mTOR signaling by sorafenib is related to induction of autophagy (Table 5, Figure 1). Most studies have shown that sorafenib is able to decrease mTORC1 phosphorylation, expression, and activity both in cultured cells *in vitro* and in xenograft tumor implantation in mice (Liu et al., 2012; Zhai et al., 2014). In HCC *in vitro* models, it has been described that sorafenib inhibits mTORC1 phosphorylation during periods from 24 to 48 h (Zhai et al., 2014; Zhang et al., 2015). Furthermore, sorafenib is able to dephosphorylate p70S6K and 4E-BP1, which are two obvious evidences of mTORC1 activity inhibition in HCC cells (Liu et al., 2012) and other cancers (Eulitt et al., 2010; Gulhati et al., 2012; Kharaziha et al., 2012; Tang et al., 2012; Eum et al., 2013; Hamed et al., 2015). However, it has been shown that sorafenib is not able to alter phosphorylation status of mTORC1 but reduces p70S6K and 4EBP1 phosphorylation in cultured non-Hodgkin lymphoma cell lines (Ramakrishnan et al., 2012). Other *in vivo* studies also suggest that sorafenib is able to reduce mTORC1 activity in HCC xenograft models followed by a decreased phosphorylation of its downstream proteins, p70S6K, 4E-BP1, and eIF-4E (Liu et al., 2012; Zhai et al., 2014). It has been shown that sorafenib reduces *in vivo* and *in vitro* mTORC1 signaling and p70S6K phosphorylation, but increases mTORC2 through increasing ^Ser2481^mTOR, being this later effect abolished by everolimus co-treatment in osterosarcoma preclinical models (Figure 1; Pignochino et al., 2013).

**Table 5:** **Effect of sorafenib on mTOR/Akt pathway in tumor cells**.

**References**	**Cell lines**	**Pathophysiological condition**	**Effect on markers of mTOR/Akt pathway**	**Global effect**
Hamed et al., 2015	GBM12	Glioblastoma multiforme	↓p-Akt ↓p-mTOR ↓p-p70	Sorafenib induces Akt/mTOR pathway inhibition when have passed only 6 h of treatment
Liu et al., 2012	PLC/PRF/5, HepG2 and Hep3B	Hepatocellular carcinoma	↓p-mTOR ↓p-p70S6K ↓p-4E-BP1 ↑p-Akt	Two hours of treatment with sorafenib is able to downregulate mTOR and all its related pathways
Ramakrishnan et al., 2012	Dohh2	Non-Hodgkin lymphoma	↓p-p70 ↓p-4E-BP1 = p-mTOR	Sorafenib fails in inactivate mTOR phosphorylation at 8 h of treatment
Tang et al., 2012	GBM15	Glioblastoma multiforme	↓p-p70 = p-mTOR	Twenty-Four hours of treatment with sorafenib reduces slightly mTOR pathway induction
Zhai et al., 2014	Huh7 and HepG2	Hepatocellular carcinoma	↓p-mTOR ↓p-p70S6K ↓p-4E-BP1 ↑p-Akt	Sorafenib inhibits mTOR pathway activity and that inactivation stimulates autophagy response
Zhang et al., 2015	SMMC-772l	Hepatocellular carcinoma	↓ Akt, PI3K and mTOR at 8 h of treatment, afterwards their levels increases	Sorafenib induces a transitory inactivation of Akt/mTOR pathway

Some researchers have assessed if the induction of autophagy by sorafenib is due to repression of mTOR signaling pathway in HCC (Shimizu et al., 2012; Zhai et al., 2015) and other cancers (Bareford et al., 2011a,b; Kharaziha et al., 2012; Eum et al., 2013). Due to dual role of autophagy in cellular death, mTOR inhibition might lead to death or survival of cancer cells, depending on whether autophagy repressed by this protein works, respectively, as a cell death inducer, or as a survival mechanism in both HCC and non-HCC cells (Kharaziha et al., 2012; Shimizu et al., 2012; Tavallai et al., 2015). Sorafenib analogs have a different ability to abolish mTOR in cultured cell lines *in vitro*. Some of them, such as regorafenib or SK-01105, are able to induce mTOR dephosphorylation similarly to sorafenib, whereas other compounds, like t-AUCMB or t-MTUCB, cannot inhibit mTOR activity, so autophagy induction mediated by those sorafenib analogs is not modulated by mTORC1 activity (Wecksler et al., 2014; Tavallai et al., 2015).

The major upstream inducer of mTORC1 pathways is PI3K/Akt signaling (Sarkar, 2013). In sorafenib-resistant cells, Akt expression is usually over-stimulated, and treatment with some specific repressors is able to increase sorafenib-related cell death, which means that combined treatment with sorafenib and an Akt inhibitor may be useful because it might improve sorafenib sensitivity (Zhai et al., 2015). Different reports have shown that sorafenib inhibits Akt activity and its phosphorylation in HCC and renal carcinoma cells (RCC) (Table 5; Eulitt et al., 2010; Gulhati et al., 2012; Serova et al., 2013; Hamed et al., 2015; Zhang et al., 2015). However, other studies found that sorafenib induces Akt phosphorylation, which will lead to generate resistance against that chemotherapeutic agent (Table 5; Liu et al., 2012; Zhai et al., 2015). Finally, a third group of works have reported that sorafenib has not influence in the phosphorylation status of Akt (Gedaly et al., 2010).

In summary, sorafenib appears to inactivate mTORC1 activity, which would contribute to autophagy induction. The mechanism responsible for those effects remains unclear, but, in some cases, could involve the PI3K/Akt pathway.

### AMPK pathway

AMPK is a heterotrimeric complex acting as a sensor of energy status in eukaryotic cells (Grahame Hardie, 2014; Novikova et al., 2015). AMPK activators may be used as adjuvants in various cancer therapies, because its stimulation can induce autophagy or cell cycle stop in tumor cell (Motoshima et al., 2006; Donadon et al., 2010; Rehman et al., 2014). Sorafenib activates AMPK being this effect potentially relevant during induction of autophagy in cancer cells (Figure 1; Eum et al., 2013; Fischer et al., 2014; Tesori et al., 2015). Sorafenib is able to induce AMPK phosphorylation in a time-dependent fashion and in a dose-dependent manner in experimental models *in vitro*, specifically in cultured cells coming from different types of human tumors (Eum et al., 2013; Fumarola et al., 2013; Pignochino et al., 2013, 2015; Fischer et al., 2014; Groenendijk et al., 2015; Tesori et al., 2015). It has been demonstrated that the incubation with 5 μM of sorafenib in multidrug resistant cells cultured *in vitro* can stimulate AMPK phosphorylation in a time-dependent manner, starting at 0.5 h after treatment (Eum et al., 2013). Sorafenib also induces AMPK phosphorylation (48 h) in lung adenocarcinoma or non-small cell lung cancer (Groenendijk et al., 2015). The drug is also able to induce AMPK activation in various breast cancer cell lines (Fumarola et al., 2013). On the other hand, there are some experimental models in which sorafenib is not able to induce AMPK phosphorylation due to the use of low concentrations of the antitumor agent (Sviripa et al., 2013). Curiously, some differences in AMPK modulation by sorafenib have been described between Huh7 and Hep3B HCC cell lines, showing increases only in Huh7 cells but without changes in Hep3B (Fischer et al., 2014).

There are two principal hypotheses which explain the mechanism by which sorafenib induces AMPK phosphorylation (Figure 1). The first one is that sorafenib generates a reduction in cellular ATP levels that increase AMP/ATP ratio and AMPK activation (Fumarola et al., 2013; Tesori et al., 2015). An experimental study which corroborates that hypothesis demonstrated that ATP level in sorafenib-treated lung adenocarcinoma cells decreases more than 50% compared to the control group (Tesori et al., 2015). On the other hand, the confirmation of the role of ROS burst during sorafenib-induced AMP activation comes from a study in which AMPK-related apoptosis was prevented by the treatment with a ROS scavenger (Pignochino et al., 2013, 2015). The activation of upstream kinases, LKB1 and CAMKKβ, is involved on sorafenib-related AMPK activation in NSCLC cells *in vitro* and *in vivo* (Groenendijk et al., 2015). Nevertheless, it seems that the AMPK pathway could be not involved in autophagy-induction by sorafenib, because LC3II expression increases sharply when AMPK is inhibited in HCC cultured cells treated with sorafenib. Whereby, it is possible that AMPK modulation by sorafenib participates in other cellular processes, different to autophagy, which can also take part into its antiproliferative action, such as apoptosis or glucose metabolism deregulation (Pignochino et al., 2013; Tesori et al., 2015).

### Endoplasmic reticulum stress

Endoplasmic reticulum is the main cellular organelle where protein synthesis, modification, and folding are carried out and where calcium is stored. In the presence of different stresses caused by physiological or pathological changes, non-folding or unfolding proteins accumulate into the organelle, generating a new condition inside the cell called ER stress. ER stress is negatively interfering with protein synthesis and affects other functions of that organelle, such as calcium homeostasis, which finally may cause programmed cell death (Tuñón et al., 2013; Kania et al., 2015). This situation leads to the activation of the unfolded protein response (UPR), which tries to recover the initial situation (Jheng et al., 2014). Some of the strategies that this response uses to alleviate ER stress are reduction of protein translation, expression of diverse chaperones, induction of protein degradation processes, such as ubiquitin-proteasome system, and degradation of portions of the endoplasmic reticulum through the autophagy pathway (Verfaillie et al., 2010). The three essential proteins in UPR induction are inositol requiring enzyme 1α (IRE1α), protein kinase R-like ER kinase (PERK) and activating transcription factor (ATF6) (Crespo et al., 2012). These macromolecules are located in basal conditions into the ER membrane and they are inhibited by the chaperone binding immunoglobulin protein (BiP). In stress situations, BiP disassembles of these molecules, causing their activation and the stimulation of three different protein cascades which promote the UPR (Malhi and Kaufman, 2011).

Sorafenib triggers the UPR response in different experimental *in vitro* models, which may contribute to the sorafenib-related induction of autophagy and to its antiproliferative effects (Table 6, Figure 1). Sorafenib-induced UPR is unrelated to MAPK inhibition because ERK repression cannot stimulate ER stress (Rahmani et al., 2007; Shi et al., 2011). More in detail, sorafenib is able to activate two of the three branches implicated in the response against ER stress (Rahmani et al., 2007; Yi et al., 2012). Specifically, this drug induces the expression of IRE1α and PERK, whereas ATF6 expression remains constant without changes in the expression on downstream proteins such as BiP (Table 6; Rahmani et al., 2007; Yi et al., 2012). In fact, it has been postulated that the effectiveness of ATF6 is consequence of the profound alteration of the secretory pathway induced by sorafenib (Yi et al., 2012). Other experiments have claimed that sorafenib reduces directly BiP expression, chaperone related with the induction of UPR mediated by this chemotherapeutic agent (Table 6; Rahmani et al., 2007; Jiang et al., 2014).

**Table 6:** **Effect of sorafenib on UPR proteins and related factors in cancerous cells**.

**References**	**Cell lines**	**Pathophysiological condition**	**Model**	**Effect on UPR-related proteins**	**Global effect on ER stress**
Dixon et al., 2014	HT-1080	Fibrosarcoma	*In vitro*	↑eIF-2α ↑ATF4 = *xbp1*	Sorafenib leads to ER stress induction
Holz et al., 2013	KM-H2, L-428, L-1236	Hodgkin lymphoma	*In vitro*	↑GADD34 ↑GADD135/CHOP ↑PERK	Sorafenib promotes ER stress and the UPR
Niessner et al., 2011	BLM; MV3, MEWO, SKMel19	Metastatic melanoma	*In vitro*	↑CHOP ↑p8	Sorafenib induces upregulation of the ER stress
Park et al., 2008	HepG2, UOK121LN,HMBC	Hepatocellular carcinoma, renal carcinoma and melanoma	*In vitro*	↑p-PERK ↑ p-eIF-2α	Sorafenib alone and sorafenib combination with vorinostat increases ER stress and autophagy in a CD95 dependent manner
Rahmani et al., 2007	K562, U937 and Jurkat	Leukemia	*In vitro*	↑p-eIF-2α ↑PERK ↑GADD34 ↑GADD135/CHOP ↓ATF6 ↓grp78/BiP = grp94 ↑IRE1 ↑slightly *xbp*1 ↑JNK1/2	Sorafenib stimulates the UPR independently of MAPK pathway inhibition
Shi et al., 2011	MHCC97-L, HepG2 and PLC/PRF/5	Hepatocellular carcinoma	*In vitro* and *in vivo*	↑IRE1, ↑p-eIF-2α ↑CHOP ↑Ca^2+^	Sorafenib induces the UPR and that generates autophagy and apoptosis stimulation on these cells
Yi et al., 2012	HepG2	Hepatocellular carcinoma	*In vitro*	↑PERK ↑*xbp1* splicing ↑*chop* ↑JNK1/2 ↑eiF2α ↑*gadd34* = ATF6a = BiP	Sorafenib activates only two of the three branches of the UPR and that increases autophagy flux

Different research has demonstrated that sorafenib induces a pronounced increase in the expression of PERK (Table 6), which modifies the activity of all its downstream proteins (Rahmani et al., 2007; Park et al., 2008; Shi et al., 2011; Yi et al., 2012; Holz et al., 2013). One of the most important is eIF2α (eukaryotic translation initiation factor 2α), is a tripartite protein complex that binds and hydrolyzes GTP during its role in recruiting the initiator methionyl-tRNA to the 40S ribosome to begin mRNA translation in eukaryotic cells (Verfaillie et al., 2010). Some studies have shown that sorafenib is able to induce eIF2α phosphorylation on Ser51 when it has passed 2 h since the start of the treatment (Rahmani et al., 2007; Yi et al., 2012; Dixon et al., 2014). The phosphorylation of eIF2α prevents binding of GTP and consequently reduces protein translation in cancer cells (Rahmani et al., 2007; Yi et al., 2012).

The last of the UPR branches which is modified by sorafenib is the IRE1α pathway. Some studies have suggested that this antitumoral agent is able to stimulate IRE1α expression, inducing its activation and changing the expression of different downstream factors (Table 6, Figure 1; Rahmani et al., 2007; Shi et al., 2011; Yi et al., 2012). In this way, IRE1α has a key role in the splicing and subsequently translation of *xbp1* (Malhi and Kaufman, 2011), and sorafenib stimulates the splicing of this gene similarly to the incubation with diverse ER stress inductors, such as tunicamycin or thapsigargin (Yi et al., 2012). Curiously, when IRE1α or *xbp1* are knocked down, cell sensibility to sorafenib increases (Rahmani et al., 2007).

The endoplasmic reticulum is the main reservoir of calcium ion (Ca^2+^) (Kania et al., 2015). Sorafenib is able to disrupt reticulum homeostasis because it discharges all the Ca^2+^ ion present in that organelle and induces its accumulation into the cytosol (Rahmani et al., 2007). This reduction of Ca^2+^endoplasmic reticulum storages increases ROS production, inducing oxidative stress, and may contribute to the cancerous cell death induced by sorafenib (Figure 1; Rahmani et al., 2007).

If ER stress is prolonged over time, UPR might be overwhelmed because that organelle may be full of unfolded and useless proteins, and that situation would generate programmed cell death through CHOP (C/EBP homologous protein) mediator, which is a transcription factor involved in the increase of the expression of a lot of genes related with apoptosis pathway (Verfaillie et al., 2010; Malhi and Kaufman, 2011). Sorafenib is able to induce the expression of CHOP and of some proteases related with ER stress programmed cell death, such as caspase-2 or -4 (Table 6). IRE1α has also been shown to induce caspase-12 oligomerization through association with TRAF2/ASK (Yoneda et al., 2001). These results reflect that UPR stimulation by that drug might be responsible, at least in a part, for sorafenib-related programmed cell death in cultured cancer cells (Rahmani et al., 2007; Niessner et al., 2011; Shi et al., 2011; Yi et al., 2012; Holz et al., 2013).

Autophagy is one of the multiple pathways induced in response to UPR upregulation, and that response might be one of the cellular mechanisms through which sorafenib induces autophagy (Fernández et al., 2015). It has been demonstrated that the repression of the IRE1α pathway leads to complete abolishment of autophagy induced by sorafenib (Figure 1; Shi et al., 2011). On the other hand, PERK pathway inhibition barely modifies the induction of autophagy by sorafenib (Shi et al., 2011). Furthermore, prolonged activation of autophagy by the drug, engulfing large ER portions with unfolded proteins and alleviating ER stress, has been related with UPR inactivation (Honma and Harada, 2013). In fact, autophagy suppression in HCC cells using pharmacological inhibitors enhances the UPR (Shi et al., 2011).

### Sphingolipids

Sphingolipids are a broad group of bioactive lipids participating in the regulation of multiple cellular routes, such as apoptosis, cell cycle, senescence, or cell differentiation (Morales et al., 2012) The most important ones are ceramides, which are a group of molecules involved in cellular death, cell cycle stop or senescence induction, and sphingosine-1-phosphate (S1P), a mediator in cell survival or cell proliferation (Hannun and Obeid, 2008). Autophagy is modulated by sphingolipid signaling, because ceramides promote lysosome and autophagosome fusion, while S1P disrupts that event. On the other hand, ceramides also promote Beclin-1 and Bcl-2 disruption and alter ER Ca^2+^ homeostasis, which generates an early induction of apoptosis (Harvald et al., 2015; Ordoñez et al., 2015). Therefore, alterations in sphingolipids balance and signaling by sorafenib might contribute to autophagy-induction mediated by that agent (Harvald et al., 2015).

The number of articles relating changes in sphingolipid generation and sorafenib effects are very few, and some of them connect the balance of those lipids with the induction of autophagy pathway (Figure 1). Sorafenib, in combination with vorinostat, is able to induce the synthesis of diverse ceramides, such as C14 or C16, mainly through of the breakdown of more complex sphingolipids by ASMase (acid sphingomyelinase), because inhibition of that enzyme generate an obviously repression in the formation of that lipids (Park et al., 2008). Nevertheless, it has been shown that *de novo* synthesis would be also relevant in ceramide generation by that drug combination (Park et al., 2010). The inhibition of both pathways into HCC cultured cells treated with sorafenib in combination with vorinostat abolishes ROS production and ER stress generation, which induces CD95 repression, and inhibition of autophagy and cell death (Park et al., 2008, 2010). In summary, ceramide formation by those chemotherapeutic drugs seems to be necessary in the induction of cell death, ER stress, ROS burden, and autophagosome formation in cultured cancer cell (Park et al., 2008, 2010). A study has also demonstrated that treatment with sorafenib decreased slightly the levels of S1P in HepG2 xenograft tumors that were grown in mice (Beljanski et al., 2011). In summary, it seems that sorafenib might have some effects on the misbalance of sphingolipid metabolism, but more studies are necessary to elucidate its specific role, and to identify the potential link with sorafenib-related autophagy induction (Figure 1).

### Beclin-1 and Bcl-2 protein family interaction

Beclin-1 is one of the most representative proteins taking part at the beginning of the autophagy pathway (Yuan et al., 2013). Recent studies have demonstrated the presence of a BH3 domain inside Beclin-1, through which it can bind to diverse antiapoptotic proteins belonging to the Bcl-2 family, like Bcl-2, Bcl-xL, or Mcl-1 (Germain et al., 2011). That interaction is able to repress Beclin-1 activity, which generates a markedly decrease on autophagy induction, being that situation reversed when Beclin-1 BH3 domain is phosphorylated, because the phosphate group prevents optimal binding between these two proteins, causing their split, Beclin-1 release and its following activation (Germain et al., 2011; Mukhopadhyay et al., 2014). There are various cellular stimuli, as starvation or oxidative stress, which alter Beclin-1 BH3 domain and, therefore, the optimal binding between that two factors (Lindqvist et al., 2014). Moreover, other proteins inside the cell with this domain, like BNIP3 or Nix, can displace Beclin-1 and Bcl-2 binding, which results in Beclin-1 release (Campello et al., 2014).

Studies on different HCC cell lines and HCC-bearing mice have shown that sorafenib treatment diminishes the interactions between Beclin-1 and Mcl-1 and disrupt the complex constituted by these proteins, inducing the release of Beclin-1 and the formation of new autophagosomes (Figure 1; Tai et al., 2013). However, interactions between Beclin-1 and other homologs of Mcl-1, such as Bcl-xL, do not undergo significant changes in autophagy induction (Tai et al., 2013). New experiments are necessary for the complete elucidation of the role of Beclin-1 in autophagy-induction mediated by sorafenib.

### miRNAs regulation

Some small non-coding RNA species can also participate in the regulation of that pathway, specifically those that belong to micro RNA (miRNA) group. These are constituted of about 20 to 24 nucleotides and its main function consists in deregulate the expression of different messenger RNAs (mRNAs) in a post-transcriptional manner (Elbashir et al., 2001; Yin and Wan, 2002). This process implies the recognition of a miRNA complementary sequence into the 3′-UTR region of the mRNA, which leads to the binding between these two molecules. miRNA is then able to inhibit mRNA translation causing, in the most of the occasions, the break of the second nucleotide chain, and the decrease of the expression of the protein which is encoded in that mRNA (Elbashir et al., 2001; Yin and Wan, 2002). The sequence that these molecules recognize in the mRNA is not exactly the complementary one, and may have some different nucleotides that prevent its perfect binding (Romaine et al., 2015). This situation implies that mRNA inhibition is very plastic because one mRNA expression can be modified by various miRNAs and vice versa, due to only one miRNA can affect and modify the translation of multiple mRNAs (Romaine et al., 2015).

There are some miRNAs involved in the regulation of the autophagy process, such as miR-224, miR-30α, miR-855-3p, miR-375, or miR-101. Their deregulation might be responsible of changes in autophagy induction when the cellular homeostasis is broken, for example, when a chemotherapeutic agent is added to cultured cancer cells (Sui et al., 2015). Some studies employing the resistant-hepatocyte rat model (R-H), which allows dissecting the different steps of hepatocarcinogenesis, showed that autophagy induction in early stage promotes the growth of preneoplastic rat liver nodules, but in late stages autophagy inhibition and miR-224 overexpression is found in neoplastic nodules when compared to normal liver (Kowalik et al., 2016). Furthermore, miR-224 upregulation has been associated to impaired autophagy in both HCC human samples and orthotopically rat models, suggesting an oncogenic role in cell migration and correlating with a poor overall survival rate in HCC (Lan et al., 2014). miR-30α is a miRNA that can repress Beclin-1 expression, which generates a reduction in autophagy activity. It has been found that its deregulation in RCC cells would interfere with the induction of autophagy flux by sorafenib and also alter sorafenib-related programmed cell death through apoptosis (Zheng et al., 2015). The knockdown of miR-30α by introducing antagomiR-30α increased Beclin-1 expression, and inhibited sorafenib-induced cytotoxicity, while following overexpression, cells become more sensitive to the drug. Therefore, the regulation of miR-30α may be crucial in the decrease of the resistance of cancerous cells to sorafenib (Figure 1; Zheng et al., 2015). Sorafenib has also been shown to induce the expression of miR-423-5p in HCC models *in vivo* and *in vitro*, and this stimulation produces a clear reduction in HCC cell proliferation and autophagy induction, suggesting that miR-423-5p could be used as a positive predictive marker of sorafenib response in HCC patients (Stiuso et al., 2015). In summary, it seems that some miRNAs are able to induce autophagy in response to sorafenib treatment, which indicates that its regulation is crucial to avoid sorafenib-related resistance in cancerous cells.

## Conclusions

Sorafenib is one of the most promising drugs for palliative treatment in HCC, but the appearance of resistant cells, and the rise of diverse side effects alter the optimal efficiency of sorafenib therapy. Therefore, a better knowledge of mechanisms contributing to sorafenib resistance or sensitivity is required for its optimal use. This review summarizes effects of sorafenib on the autophagic process in HCC cells, and the influence of autophagy on sorafenib-related cell resistance generation. This agent, alone or in combination with other drugs, antioxidants, or natural compounds is able to induce autophagy, causing either cellular death or survival, which mainly depends on the complex integrative crosstalk of different intracellular signals. Sorafenib-related autophagy will generate cell drug resistance if it induces the survival of cancer cells making them insensitive to death stimulus. On the other hand, there are diverse cellular pathways, protein interactions or nucleic acid molecules able to alter the range of autophagy induction in response to sorafenib treatment. Hence, changes in their activation may be useful to increase sensitivity to sorafenib. Some intracellular pathways such as mTOR pathway, UPR response, sphingolipid generation, and the critical interaction of Bcl-2 and Mcl-1 with Beclin-1, as well as changes in the miRNA cellular pattern are essential in the final regulatory outcome of autophagy. Meanwhile, AMPK induction fails to induce sorafenib-related autophagy. Further research is needed to elucidate the role of autophagy in tumor cell resistance to sorafenib. These studies could lead to the increase of the drug effectiveness, reducing the doses of sorafenib in the treatment of cancer diseases, or to the development of new sorafenib analogs with less side effects and improved antiproliferative effects.

## Author contributions

NP, JM, and JG conceived and designed the manuscript. All authors contributed to the writing.

### Conflict of interest statement

The authors declare that the research was conducted in the absence of any commercial or financial relationships that could be construed as a potential conflict of interest.

## References

[B1] Abdel-Rahman O. Fouad M. (2014). Sorafenib-based combination as a first line treatment for advanced hepatocellular carcinoma: a systematic review of the literature. Crit. Rev. Oncol. Hematol. 91, 1–8. 10.1016/j.critrevonc.2013.12.013 24457121

[B2] Adnane L. Trail P. A. Taylor I. Wilhelm S. M. (2006). Sorafenib (BAY 43-9006, Nexavar), a dual-action inhibitor that targets RAF/MEK/ERK pathway in tumor cells and tyrosine kinases VEGFR/PDGFR in tumor vasculature. Methods Enzymol. 407, 597–612. 10.1016/S0076-6879(05)07047-3 16757355

[B3] Alonso-Gordoa T. Diez J. J. Duran M. Grande E. Díez J. J. Durán M.. (2015). Advances in thyroid cancer treatment: latest evidence and clinical potential. Ther. Adv. Med. Oncol. 7, 22–38. 10.1177/1758834014551936 25553081 PMC4265091

[B4] Antar A. Kharfan-Dabaja M. A. Mahfouz R. Bazarbachi A. (2014). Sorafenib maintenance appears safe and improves clinical outcomes in FLT3-ITD acute myeloid leukemia after allogeneic hematopoietic cell transplantation. Clin. Lymphoma. Myeloma Leuk. 15, 298–302. 10.1016/j.clml.2014.12.005 25550214

[B5] Bareford M. D. Hamed H. A. Tang Y. Cruickshanks N. Burow M. E. Fisher P. B.. (2011a). Sorafenib enhances pemetrexed cytotoxicity through an autophagy-dependent mechanism in cancer cells. Autophagy 7, 1261–1262. 10.4161/auto.7.10.17029 21814046 PMC3210312

[B6] Bareford M. D. Park M. A. Yacoub A. Hamed H. A. Tang Y. Cruickshanks N.. (2011b). Sorafenib enhances pemetrexed cytotoxicity through an autophagy-dependent mechanism in cancer cells. Cancer Res. 71, 4955–4967. 10.1158/0008-5472.CAN-11-0898 21622715 PMC3139015

[B7] Beljanski V. Lewis C. S. Smith C. D. (2011). Antitumor activity of sphingosine kinase 2 inhibitor ABC294640 and sorafenib in hepatocellular carcinoma xenografts. Cancer Biol. Ther. 11, 524–534. 10.4161/cbt.11.5.14677 21258214 PMC3087901

[B8] Booth L. Roberts J. L. Cruickshanks N. Conley A. Durrant D. E. Das A.. (2014). Phosphodiesterase 5 inhibitors enhance chemotherapy killing in gastrointestinal/genitourinary cancer cells. Mol. Pharmacol. 85, 408–419. 10.1124/mol.113.090043 24353313 PMC3935155

[B9] Campello S. Strappazzon F. Cecconi F. (2014). Mitochondrial dismissal in mammals, from protein degradation to mitophagy. Biochim. Biophys. Acta Bioenerg. 1837, 451–460. 10.1016/j.bbabio.2013.11.010 24275087

[B10] Carr B. I. Cavallini A. Lippolis C. D'Alessandro R. Messa C. Refolo M. G.. (2013). Fluoro-Sorafenib (Regorafenib) effects on hepatoma cells: growth inhibition, quiescence, and recovery. J. Cell. Physiol. 228, 292–297. 10.1002/jcp.24148 22777740 PMC4509637

[B11] Cervello M. Bachvarov D. Lampiasi N. Cusimano A. Azzolina A. McCubrey J. A.. (2012). Molecular mechanisms of sorafenib action in liver cancer cells. Cell Cycle 11, 2843–2855. 10.4161/cc.21193 22801548

[B12] Chen K. F. Tai W. T. Liu T. H. Huang H. P. Lin Y. C. Shiau C. W.. (2010). Sorafenib overcomes TRAIL resistance of hepatocellular carcinoma cells through the inhibition of STAT3. Clin. Cancer Res. 16, 5189–5199. 10.1158/1078-0432.CCR-09-3389 20884624

[B13] Chi K. N. Ellard S. L. Hotte S. J. Czaykowski P. Moore M. Ruether J. D.. (2008). A phase II study of sorafenib in patients with chemo-naive castration-resistant prostate cancer. Ann. Oncol. 19, 746–751. 10.1093/annonc/mdm554 18056648

[B14] Chiou J. F. Tai C. J. Huang M. T. Wei P. L. Wang Y. H. An J.. (2010). Glucose-regulated protein 78 is a novel contributor to acquisition of resistance to sorafenib in hepatocellular carcinoma. Ann. Surg. Oncol. 17, 603–612. 10.1245/s10434-009-0718-8 19830497

[B15] Choi K. S. (2012). Autophagy and cancer. Exp. Mol. Med. 44, 109–120. 10.3858/emm.2012.44.2.033 22257886 PMC3296807

[B16] Crespo I. San-Miguel B. Prause C. Marroni N. Cuevas M. J. González-Gallego J.. (2012). Glutamine Treatment Attenuates Endoplasmic Reticulum Stress and Apoptosis in TNBS-Induced Colitis. PLoS ONE 7:E50407. 10.1371/journal.pone.0050407 23209735 PMC3508929

[B17] Das A. Durrant D. Salloum F. N. Xi L. Kukreja R. C. (2015). PDE5 inhibitors as therapeutics for heart disease, diabetes and cancer. Pharmacol. Ther. 147, 12–21. 10.1016/j.pharmthera.2014.10.003 25444755 PMC4494657

[B18] Dixon S. J. Patel D. N. Welsch M. Skouta R. Lee E. D. Hayano M.. (2014). Pharmacological inhibition of cystine-glutamate exchange induces endoplasmic reticulum stress and ferroptosis. Elife 3:e02523. 10.7554/eLife.02523 24844246 PMC4054777

[B19] Dokmanovic M. Clarke C. Marks P. A. (2007). Histone deacetylase inhibitors: overview and perspectives. Mol. Cancer Res. 5, 981–989. 10.1158/1541-7786.MCR-07-0324 17951399

[B20] Donadon V. Balbi M. Mas M. D. Casarin P. Zanette G. (2010). Metformin and reduced risk of hepatocellular carcinoma in diabetic patients with chronic liver disease. Liver Int. 30, 750–758. 10.1111/j.1478-3231.2010.02223.x 20331505

[B21] Elbashir S. M. Harborth J. Lendeckel W. Yalcin A. Weber K. Tuschi T. (2001). Duplexes of 21-nucleotide RNAs mediate RNA interference in differentiated mouse ES cells. Nature 411, 494–498. 10.1038/35078107 11373684

[B22] Erber R. Thurnher A. Katsen A. D. Groth G. Kerger H. Hammes H. P.. (2004). Combined inhibition of VEGF and PDGF signaling enforces tumor vessel regression by interfering with pericyte-mediated endothelial cell survival mechanisms. FASEB J. 18, 338–340. 10.1096/fj.03-0271fje 14657001

[B23] Escudier B. Eisen T. Stadler W. M. Szczylik C. Oudard S. Siebels M.. (2007). Sorafenib in advanced clear-cell renal-cell carcinoma. N. Engl. J. Med. 356, 125–134. 10.4161/cbt.10.12.13497 17215530

[B24] Eskelinen E. L. (2011). The dual role of autophagy in cancer. Curr. Opin. Pharmacol. 11, 294–300. 10.1016/j.coph.2011.03.009 21498118

[B25] Eulitt P. J. Park M. A. Hossein H. Cruikshanks N. Yang C. Dmitriev I. P.. (2010). Enhancing mda-7/IL-24 therapy in renal carcinoma cells by inhibiting multiple protective signaling pathways using sorafenib and by Ad.5/3 gene delivery. Cancer Biol. Ther. 10, 1290–1305. 10.4161/cbt.10.12.13497 20948318 PMC3047088

[B26] Eum K. H. Ahn S. K. Kang H. Lee M. (2013). Differential inhibitory effects of two Raf-targeting drugs, sorafenib and PLX4720, on the growth of multidrug-resistant cells. Mol. Cell. Biochem. 372, 65–74. 10.1007/s11010-012-1446-0 22941213

[B27] Ferlay J. Soerjomataram I. Dikshit R. Eser S. Mathers C. Rebelo M.. (2015). Cancer incidence and mortality worldwide: sources, methods and major patterns in GLOBOCAN 2012. Int. J. Cancer 136, E359–E386. 10.1002/ijc.29210 25220842

[B28] Fernández A. Ordóñez R. Reiter R. J. González-Gallego J. Mauriz J. L. (2015). Melatonin and endoplasmic reticulum stress: relation to autophagy and apoptosis. J. Pineal Res. 59, 292–307. 10.1111/jpi.12264 26201382

[B29] Fischer T. D. Wang J. H. Vlada A. Kim J. S. Behrns K. E. (2014). Role of autophagy in differential sensitivity of hepatocarcinoma cells to sorafenib. World J. Hepatol. 6, 752–758. 10.4254/wjh.v6.i10.752 25349646 PMC4209420

[B30] Forner A. Llovet J. M. Bruix J. (2012). Hepatocellular carcinoma. Lancet 379, 1245–1255. 10.1016/S0140-6736(11)61347-0 22353262 PMC13537732

[B31] Fumarola C. Caffarra C. La Monica S. Galetti M. Alfieri R. R. Cavazzoni A.. (2013). Effects of sorafenib on energy metabolism in breast cancer cells: role of AMPK-mTORC1 signaling. Breast Cancer Res. Treat. 141, 67–78. 10.1007/s10549-013-2668-x 23963659

[B32] Gauthier A. Ho M. (2013). Role of sorafenib in the treatment of advanced hepatocellular carcinoma: an update. Hepatol. Res. 43, 147–154. 10.1111/j.1872-034X.2012.01113.x 23145926 PMC3574194

[B33] Gedaly R. Angulo P. Hundley J. Daily M. F. Chen C. Koch A.. (2010). PI-103 and sorafenib inhibit hepatocellular carcinoma cell proliferation by blocking Ras/Raf/MAPK and PI3K/AKT/mTOR pathways. Anticancer Res. 30, 4951–4958. 21187475 PMC3141822

[B34] Germain M. Nguyen A. P. Le Grand J. N. Arbour N. Vanderluit J. L. Park D. S.. (2011). MCL-1 is a stress sensor that regulates autophagy in a developmentally regulated manner. EMBO J. 30, 395–407. 10.1038/emboj.2010.327 21139567 PMC3025469

[B35] Giannini G. Cabri W. Fattorusso C. Rodriquez M. (2012). Histone deacetylase inhibitors in the treatment of cancer: overview and perspectives. Future Med. Chem. 4, 1439–1460. 10.4155/fmc.12.80 22857533

[B36] Gollob J. A. Rathmell W. K. Richmond T. M. Marino C. B. Miller E. K. Grigson G.. (2007). Phase II trial of sorafenib plus interferon alfa-2β as first- or second-line therapy in patients with metastatic renal cell cancer. J. Clin. Oncol. 25, 3288–3295. 10.1200/JCO.2007.10.8613 17664476

[B37] Gonzalez S. A. (2014). Novel biomarkers for hepatocellular carcinoma surveillance: has the future arrived? Hepatobiliary Surg. Nutr. 3, 410–414. 10.3978/j.issn.2304-3881.2014.07.06 25568864 PMC4273110

[B38] Grahame Hardie D. (2014). AMP-activated protein kinase: a key regulator of energy balance with many roles in human disease. J. Intern. Med. 276, 543–559. 10.1111/joim.12268 24824502 PMC5705060

[B39] Groenendijk F. H. Mellema W. W. van der Burg E. Schut E. Hauptmann M. Horlings H. M.. (2015). Sorafenib synergizes with metformin in NSCLC through AMPK pathway activation. Int. J. Cancer 136, 1434–1444. 10.1002/ijc.29113 25080865 PMC4312923

[B40] Guan Y. S. He Q. (2011). Sorafenib: activity and clinical application in patients with hepatocellular carcinoma. Expert. Opin. Pharmacother. 12, 303–313. 10.1517/14656566.2011.546346 21226640

[B41] Guidetti A. Carlo-Stella C. Locatelli S. L. Malorni W. Pierdominici M. Barbati C.. (2012). Phase II study of sorafenib in patients with relapsed or refractory lymphoma. Br. J. Haematol. 158, 108–119. 10.1111/j.1365-2141.2012.09139.x 22571717

[B42] Gulhati P. Zaytseva Y. Y. Valentino J. D. Stevens P. D. Kim J. T. Sasazuki T.. (2012). Sorafenib enhances the therapeutic efficacy of rapamycin in colorectal cancers harboring oncogenic KRAS and PIK3CA. Carcinogenesis 33, 1782–1790. 10.1093/carcin/bgs203 22696593 PMC3514899

[B43] Hamed H. A. Tavallai S. Grant S. Poklepovic A. Dent P. (2015). Sorafenib/regorafenib and lapatinib interact to kill CNS tumor cells. J. Cell. Physiol. 230, 131–139. 10.1002/jcp.24689 24911215 PMC4182138

[B44] Hannun Y. A. Obeid L. M. (2008). Principles of bioactive lipid signalling: lessons from sphingolipids. Nat. Rev. Cell Biol. 9, 139–150. 10.1038/nrm2329 18216770

[B45] Harvald E. B. Olsen A. S. Faergeman N. J. (2015). Autophagy in the light of sphingolipid metabolism. Apoptosis 20, 658–670. 10.1007/s10495-015-1108-2 25682163 PMC4376959

[B46] He C. Klionsky D. J. (2009). Regulation mechanisms and signaling pathways of autophagy. Annu. Rev. Genet. 43, 67–93. 10.1146/annurev-genet-102808-114910 19653858 PMC2831538

[B47] Holz M. S. Janning A. Renné C. Gattenlöhner S. Spieker T. Bräuninger A. (2013). Induction of endoplasmic reticulum stress by sorafenib and activation of NF-κB by lestaurtinib as a novel resistance mechanism in Hodgkin lymphoma cell lines. Mol. Cancer Ther. 12, 173–183. 10.1158/1535-7163.mct-12-0532 23243060

[B48] Honma Y. Harada M. (2013). Sorafenib enhances proteasome inhibitor-mediated cytotoxicity via inhibition of unfolded protein response and keratin phosphorylation. Exp. Cell Res. 319, 2166–2178. 10.1016/j.yexcr.2013.05.023 23727131

[B49] Jakubowicz-Gil J. Langner E. Badziul D. Wertel I. Rzeski W. (2014). Quercetin and sorafenib as a novel and effective couple in programmed cell death induction in human gliomas. Neurotox. Res. 26, 64–77. 10.1007/s12640-013-9452-x 24366851 PMC4035551

[B50] Jheng J. R. Ho J. Y. Horng J. T. (2014). ER stress, autophagy, and RNA viruses. Front. Microbiol. 5:388. 10.3389/fmicb.2014.00388 25140166 PMC4122171

[B51] Jiang X. Kanda T. Nakamoto S. Miyamura T. Wu S. Yokosuka O. (2014). Involvement of androgen receptor and glucose-regulated protein 78 kDa in human hepatocarcinogenesis. Exp. Cell Res. 323, 326–336. 10.1016/j.yexcr.2014.02.017 24583399

[B52] Kania E. Pajak B. Orzechowski A. (2015). Calcium homeostasis and ER stress in control of autophagy in cancer cells. Biomed Res. Int. 2015:352794. 10.1155/2015/352794 25821797 PMC4363509

[B53] Kharaziha P. Ceder S. Sanchez C. Panaretakis T. (2013). Multitargeted therapies for multiple myeloma. Autophagy 9, 255–257. 10.4161/auto.22738 23183549 PMC3552894

[B54] Kharaziha P. De Raeve H. Fristedt C. Li Q. Gruber A. Johnsson P.. (2012). Sorafenib has potent antitumor activity against multiple myeloma *in vitro, ex vivo*, and *in vivo* in the 5T33MM mouse model. Cancer Res. 72, 5348–5362. 10.1158/0008-5472.CAN-12-0658 22952216

[B55] Knievel J. Schulz W. A. Greife A. Hader C. Lubke T. Schmitz I.. (2014). Multiple mechanisms mediate resistance to sorafenib in urothelial cancer. Int. J. Mol. Sci. 15, 20500–20517. 10.3390/ijms151120500 25387078 PMC4264180

[B56] Kowalik M. A. Perra A. Ledda-Columbano G. M. Ippolito G. Piacentini M. Columbano A.. (2016). Induction of autophagy promotes the growth of early preneoplastic rat liver nodules. Oncotarget 7, 5788–5799. 10.18632/oncotarget.6810 26735341 PMC4868721

[B57] Kummar S. Copur M. S. Rose M. Wadler S. Stephenson J. O'Rourke M.. (2011). A phase I study of the chinese herbal medicine PHY906 as a modulator of irinotecan-based chemotherapy in patients with advanced colorectal cancer. Clin. Colorectal Cancer 10, 85–96. 10.1016/j.clcc.2011.03.003 21859559

[B58] Lam W. Jiang Z. Guan F. Huang X. Hu R. Wang J.. (2015). PHY906(KD018), an adjuvant based on a 1800-year-old Chinese medicine, enhanced the anti-tumor activity of Sorafenib by changing the tumor microenvironment. Sci. Rep. 5:9384. 10.1038/srep09384 25819872 PMC4377583

[B59] Lan S.-H. Wu S.-Y. Zuchini R. Lin X.-Z. Su I.-J. Tsai T.-F.. (2014). Autophagy suppresses tumorigenesis of hepatitis B virus-associated hepatocellular carcinoma through degradation of microRNA-224. Hepatology 59, 505–517. 10.1002/hep.26659 23913306 PMC4298796

[B60] Li Y. Li S. Qin X. Hou W. Dong H. Yao L.. (2014). The pleiotropic roles of sphingolipid signaling in autophagy. Cell Death Dis. 5, e1245. 10.1038/cddis.2014.215 24853423 PMC4047895

[B61] Lian J. Ni Z. Dai X. Su C. Smith A. R. Xu L.. (2012). Sorafenib sensitizes (-)-gossypol-induced growth suppression in androgen-independent prostate cancer cells via Mcl-1 inhibition and Bak activation. Mol. Cancer Ther. 11, 416–426. 10.1158/1535-7163.MCT-11-0559 22188816

[B62] Lin C. I. Whang E. E. Lorch J. H. Ruan D. T. (2012). Autophagic activation potentiates the antiproliferative effects of tyrosine kinase inhibitors in medullary thyroid cancer. Surgery 152, 1142–1149. 10.1016/j.surg.2012.08.016 23158184

[B63] Lin J.-C. Liu C.-L. Lee J.-J. Liu T.-P. Ko W.-C. Hung Y. C.. (2013). Sorafenib induces autophagy and suppresses activation of human macrophage. Int. Immunopharmacol. 15, 333–339. 10.1016/j.intimp.2013.01.006 23337882 PMC7106104

[B64] Lindqvist L. M. Heinlein M. Huang D. C. Vaux D. L. (2014). Prosurvival Bcl-2 family members affect autophagy only indirectly, by inhibiting Bax and Bak. Proc. Natl. Acad. Sci. U.S.A. 111, 8512–8517. 10.1073/pnas.1406425111 24912196 PMC4060681

[B65] Liu J. Fan L. Wang H. Sun G. (2016). Autophagy, a double-edged sword in anti-angiogenesis therapy. Med. Oncol. 33, 1–13. 10.1007/s12032-015-0721-9 26715036

[B66] Liu L. Cao Y. Chen C. Zhang X. McNabola A. Wilkie D.. (2006). Sorafenib blocks the RAF/MEK/ERK pathway, inhibits tumor angiogenesis, and induces tumor cell apoptosis in hepatocellular carcinoma model PLC/PRF/5. Cancer Res. 66, 11851–11858. 10.1158/0008-5472.CAN-06-1377 17178882

[B67] Liu L. Ho R. L. K. Chen G. G. Lai P. B. S. (2012). Sorafenib inhibits hypoxia-inducible factor-1α synthesis: implications for antiangiogenic activity in hepatocellular carcinoma. Clin. Cancer Res. 18, 5662–5671. 10.1158/1078-0432.CCR-12-0552 22929805

[B68] Liu S. H. Cheng Y. C. (2012). Old formula, new Rx: the journey of PHY906 as cancer adjuvant therapy. J. Ethnopharmacol. 140, 614–623. 10.1016/j.jep.2012.01.047 22326673

[B69] Llovet J. M. Ricci S. Mazzaferro V. Hilgard P. Gane E. Blanc J. F.. (2008). Sorafenib in advanced hepatocellular carcinoma. N. Engl. J. Med. 359, 378–390. 10.1056/NEJMoa0708857 18650514

[B70] Luo Y. Shi Y. Xing P. Wang L. Feng Y. Han X.. (2014). Sorafenib in metastatic radioactive iodine-refractory differentiated thyroid cancer: a pilot study. Mol. Clin. Oncol. 2, 87–92. 10.3892/mco.2013.199 24649313 PMC3916210

[B71] Malhi H. Kaufman R. J. (2011). Endoplasmic reticulum stress in liver disease. J. Hepatol. 54, 795–809. 10.1016/j.jhep.2010.11.005 21145844 PMC3375108

[B72] Manov I. Pollak Y. Broneshter R. Iancu T. C. (2011). Inhibition of doxorubicin-induced autophagy in hepatocellular carcinoma Hep3B cells by sorafenib–the role of extracellular signal-regulated kinase counteraction. FEBS J. 278, 3494–3507. 10.1111/j.1742-4658.2011.08271.x 21790999

[B73] Manwani B. McCullough L. D. (2013). Function of the master energy regulator adenosine monophosphate-activated protein kinase in stroke. J. Neurosci. Res. 91, 1018–1029. 10.1002/jnr.23207 23463465 PMC4266469

[B74] Martin A. P. Park M. A. Mitchell C. Walker T. Rahmani M. Thorburn A.. (2009). BCL-2 family inhibitors enhance histone deacetylase inhibitor and sorafenib lethality via autophagy and overcome blockade of the extrinsic pathway to facilitate killing. Mol. Pharmacol. 76, 327–341. 10.1124/mol.109.056309 19483105 PMC2713125

[B75] Mizushima N. Komatsu M. (2011). Autophagy: renovation of cells and tissues. Cell 147, 728–741. 10.1016/j.cell.2011.10.026 22078875

[B76] Mizushima N. (2010). The role of the Atg1/ULK1 complex in autophagy regulation. Curr. Opin. Cell Biol. 22, 132–139. 10.1016/j.ceb.2009.12.004 20056399

[B77] Morales A. Mari M. Garcia-Ruiz C. Colell A. Fernandez-Checa J. C. (2012). Hepatocarcinogenesis and ceramide/cholesterol metabolism. Anticancer Agents Med. Chem. 12, 364–375. 10.2174/187152012800228689 22043999

[B78] Motoshima H. Goldstein B. J. Igata M. Araki E. (2006). AMPK and cell proliferation–AMPK as a therapeutic target for atherosclerosis and cancer. J. Physiol. 574, 63–71. 10.1113/jphysiol.2006.108324 16613876 PMC1817805

[B79] Mukhopadhyay S. Panda P. K. Sinha N. Das D. N. Bhutia S. K. (2014). Autophagy and apoptosis: where do they meet? Apoptosis 19, 555–566. 10.1007/s10495-014-0967-2 24415198

[B80] Neufeld T. P. (2010). TOR-dependent control of autophagy: biting the hand that feeds. Curr. Opin. Cell Biol. 22, 157–168. 10.1016/j.ceb.2009.11.005 20006481 PMC2854204

[B81] Niessner H. Beck D. Sinnberg T. Lasithiotakis K. Maczey E. Gogel J.. (2011). The farnesyl transferase inhibitor lonafarnib inhibits mTOR signaling and enforces sorafenib-induced apoptosis in melanoma cells. J. Invest. Dermatol. 131, 468–479. 10.1038/jid.2010.297 20944654

[B82] Novikova D. S. Garabadzhiu A. V. Melino G. Barlev N. A. Tribulovich V. G. (2015). AMP-activated protein kinase: structure, function, and role in pathological processes. Biochemistry 80, 127–144. 10.1134/S0006297915020017 25756529

[B83] Ordoñez R. Fernández A. Prieto-Domínguez N. Martínez L. García-Ruiz C. Fernández-Checa J. C.. (2015). Ceramide metabolism regulates autophagy and apoptotic-cell death induced by melatonin in liver cancer cells. J. Pineal Res. 59, 178–189. 10.1111/jpi.12249 25975536 PMC4523438

[B84] Orsi A. Polson H. E. Tooze S. A. (2010). Membrane trafficking events that partake in autophagy. Curr. Opin. Cell Biol. 22, 150–156. 10.1016/j.ceb.2009.11.013 20036114

[B85] Park M. A. Reinehr R. Haussinger D. Voelkel-Johnson C. Ogretmen B. Yacoub A.. (2010). Sorafenib activates CD95 and promotes autophagy and cell death via Src family kinases in gastrointestinal tumor cells. Mol. Cancer Ther. 9, 2220–2231. 10.1158/1535-7163.MCT-10-0274 20682655 PMC2933415

[B86] Park M. A. Zhang G. Martin A. P. Hamed H. Mitchell C. Hylemon P. B.. (2008). Vorinostat and sorafenib increase ER stress, autophagy and apoptosis via ceramide-dependent CD95 and PERK activation. Cancer Biol. Ther. 7, 1648–1662. 10.4161/cbt.7.10.6623 18787411 PMC2674577

[B87] Pfisterer S. G. Mauthe M. Codogno P. Proikas-Cezanne T. (2011). Ca2+/calmodulin-dependent kinase (CaMK) signaling via CaMKI and AMP-activated protein kinase contributes to the regulation of WIPI-1 at the onset of autophagy. Mol. Pharmacol. 80, 1066–1075. 10.1124/mol.111.071761 21896713

[B88] Pignochino Y. Dell'Aglio C. Basirico M. Capozzi F. Soster M. Marchio S.. (2013). The combination of sorafenib and everolimus abrogates mTORC1 and mTORC2 upregulation in osteosarcoma preclinical models. Clin. Cancer Res. 19, 2117–2131. 10.1158/1078-0432.CCR-12-2293 23434734

[B89] Pignochino Y. Dell'Aglio C. Inghilleri S. Zorzetto M. Basirico M. Capozzi F.. (2015). The combination of sorafenib and everolimus shows antitumor activity in preclinical models of malignant pleural mesothelioma. BMC Cancer 15:374. 10.1186/s12885-015-1363-1 25952930 PMC4429519

[B90] Rahmani M. Davis E. M. Bauer C. Dent P. Grant S. (2005). Apoptosis induced by the kinase inhibitor BAY 43-9006 in human leukemia cells involves down-regulation of Mcl-1 through inhibition of translation. J. Biol. Chem. 280, 35217–35227. 10.1074/jbc.M506551200 16109713

[B91] Rahmani M. Davis E. M. Crabtree T. R. Habibi J. R. Nguyen T. K. Dent P.. (2007). The kinase inhibitor sorafenib induces cell death through a process involving induction of endoplasmic reticulum stress. Mol. Cell. Biol. 27, 5499–5513. 10.1128/MCB.01080-06 17548474 PMC1952105

[B92] Ramakrishnan V. Timm M. Haug J. L. Kimlinger T. K. Halling T. Wellik L. E.. (2012). Sorafenib, a multikinase inhibitor, is effective *in vitro* against non-Hodgkin lymphoma and synergizes with the mTOR inhibitor rapamycin. Am. J. Hematol. 87, 277–283. 10.1002/ajh.22263 22190165 PMC3465673

[B93] Rehman G. Shehzad A. Khan A. L. Hamayun M. (2014). Role of AMP-activated protein kinase in cancer therapy. Arch. Pharm. (Weinheim). 347, 457–468. 10.1002/ardp.201300402 24677093

[B94] Rockwell S. Grove T. A. Liu Y. Cheng Y. C. Higgins S. A. Booth C. J. (2013). Preclinical studies of the Chinese Herbal Medicine formulation PHY906 (KD018) as a potential adjunct to radiation therapy. Int. J. Radiat. Biol. 89, 16–25. 10.3109/09553002.2012.717733 22856538 PMC3660854

[B95] Romaine S. P. Tomaszewski M. Condorelli G. Samani N. J. (2015). MicroRNAs in cardiovascular disease: an introduction for clinicians. Heart 101, 921–928. 10.1136/heartjnl-2013-305402 25814653 PMC4484262

[B96] Rossi L. Zoratto F. Papa A. Iodice F. Minozzi M. Frati L.. (2010). Current approach in the treatment of hepatocellular carcinoma. World J. Gastrointest. Oncol. 2, 348–359. 10.4251/wjgo.v2.i9.348 21160806 PMC2999141

[B97] Sakai K. Takeda H. Nishijima N. Orito E. Joko K. Uchida Y.. (2015). Targeted DNA and RNA sequencing of fine-needle biopsy FFPE specimens in patients with unresectable hepatocellular carcinoma treated with sorafenib. Oncotarget 6, 21636–21644. 10.18632/oncotarget.4270 26046304 PMC4673292

[B98] San-Miguel B. Crespo I. Sánchez D. I. González-Fernández B. Ortiz de Urbina J. J. Tuñón M. J.. (2015). Melatonin inhibits autophagy and endoplasmic reticulum stress in mice with carbon tetrachloride-induced fibrosis. J. Pineal Res. 59, 151–162. 10.1111/jpi.12247 25958928

[B99] San-Miguel B. Crespo I. Vallejo D. Alvarez M. Prieto J. González-Gallego J.. (2014). Melatonin modulates the autophagic response in acute liver failure induced by the rabbit hemorrhagic disease virus. J. Pineal Res. 56, 313–321. 10.1111/jpi.12124 24499270 PMC7166588

[B100] Sarkar S. (2013). Regulation of autophagy by mTOR-dependent and mTOR-independent pathways: autophagy dysfunction in neurodegenerative diseases and therapeutic application of autophagy enhancers. Biochem. Soc. Trans. 41, 1103–1130. 10.1042/BST20130134 24059496

[B101] Scarlatti F. Bauvy C. Ventruti A. Sala G. Cluzeaud F. Vandewalle A.. (2004). Ceramide-mediated macroautophagy involves inhibition of protein kinase B and up-regulation of beclin 1. J. Biol. Chem. 279, 18384–18391. 10.1074/jbc.M313561200 14970205

[B102] Sengupta S. Peterson T. R. Sabatini D. M. (2010). Regulation of the mTOR complex 1 pathway by nutrients, growth factors, and stress. Mol. Cell 40, 310–322. 10.1016/j.molcel.2010.09.026 20965424 PMC2993060

[B103] Serova M. de Gramont A. Tijeras-Raballand A. Dos Santos C. Riveiro M. E. Slimane K.. (2013). Benchmarking effects of mTOR, PI3K, and dual PI3K/mTOR inhibitors in hepatocellular and renal cell carcinoma models developing resistance to sunitinib and sorafenib. Cancer Chemother. Pharmacol. 71, 1297–1307. 10.1007/s00280-013-2129-6 23479136

[B104] Shi Y.-H. H. Ding Z.-B. B. Zhou J. Hui B. Shi G.-M. M. Ke A.-W. W.. (2011). Targeting autophagy enhances sorafenib lethality for hepatocellular carcinoma via ER stress-related apoptosis. Autophagy 7, 1159–1172. 10.4161/auto.7.10.16818 21691147

[B105] Shimizu S. Takehara T. Hikita H. Kodama T. Tsunematsu H. Miyagi T.. (2012). Inhibition of autophagy potentiates the antitumor effect of the multikinase inhibitor sorafenib in hepatocellular carcinoma. Int. J. Cancer. J. 131, 548–557. 10.1002/ijc.26374 21858812

[B106] Sou Y. S. Waguri S. Iwata J. Ueno T. Fujimura T. Hara T.. (2008). The Atg8 conjugation system is indispensable for proper development of autophagic isolation membranes in mice. Mol. Biol. Cell 19, 4762–4775. 10.1091/mbc.E08-03-0309 18768753 PMC2575156

[B107] Stiuso P. Potenza N. Lombardi A. Ferrandino I. Monaco A. Zappavigna S.. (2015). MicroRNA-423-5p promotes autophagy in cancer cells and is increased in serum from hepatocarcinoma patients treated with sorafenib. Mol. Ther. Acids 4, e233. 10.1038/mtna.2015.8 25782064

[B108] Sui X. Zhu J. Zhou J. Wang X. Li D. Han W.. (2015). Epigenetic modifications as regulatory elements of autophagy in cancer. Cancer Lett. 360, 106–113. 10.1016/j.canlet.2015.02.009 25687886

[B109] Sun K. Guo X.-L. Zhao Q. Jing Y. Kou X. Xie X.. (2013). Paradoxical role of autophagy in the dysplastic and tumor-forming stages of hepatocarcinoma development in rats. Cell Death Dis. 4, e501. 10.1038/cddis.2013.35 23429287 PMC3734842

[B110] Sviripa V. Zhang W. Conroy M. D. Schmidt E. S. Liu A. X. Truong J.. (2013). Fluorinated, N,N'-diarylureas as AMPK activators. Bioorg. Med. Chem. Lett. 23, 1600–1603. 10.1016/j.bmcl.2013.01.096 23414799 PMC3594501

[B111] Tai W. T. Shiau C. W. Chen H. L. Liu C. Y. Lin C. S. Cheng A. L.. (2013). Mcl-1-dependent activation of Beclin 1 mediates autophagic cell death induced by sorafenib and SC-59 in hepatocellular carcinoma cells. Cell Death Dis. 4, e485. 10.1038/cddis.2013.18 23392173 PMC3734819

[B112] Tang Y. Yacoub A. Hamed H. A. Poklepovic A. Tye G. Grant S.. (2012). Sorafenib and HDAC inhibitors synergize to kill CNS tumor cells. Cancer Biol. Ther. 13, 567–574. 10.4161/cbt.19771 22406992 PMC3679096

[B113] Tanida I. (2011). Autophagy basics. Microbiol. Immunol. 55, 1–11. 10.1111/j.1348-0421.2010.00271.x 21175768

[B114] Tavallai M. Hamed H. A. Roberts J. L. Cruickshanks N. Chuckalovcak J. Poklepovic A.. (2015). Nexavar/stivarga and viagra interact to kill tumor cells. J. Cell. Physiol. 230, 2281–2298. 10.1002/jcp.24961 25704960 PMC4835179

[B115] Tesori V. Piscaglia A. C. Samengo D. Barba M. Bernardini C. Scatena R.. (2015). The multikinase inhibitor Sorafenib enhances glycolysis and synergizes with glycolysis blockade for cancer cell killing. Sci. Rep. 5:9149. 10.1038/srep09149 25779766 PMC4361992

[B116] Tian Y. Kuo C. Sir D. Wang L. Govindarajan S. Petrovic L. M.. (2015). Autophagy inhibits oxidative stress and tumor suppressors to exert its dual effect on hepatocarcinogenesis. Cell Death Differ. 22, 1025–1034. 10.1038/cdd.2014.201 25526090 PMC4423188

[B117] Togashi Y. Nishio K. (2015). Kinase inhibitors and their resistance. Nihon Rinsho. 73, 1323–1329. 26281685

[B118] Torre L. A. Bray F. Siegel R. L. Ferlay J. Lortet-Tieulent J. Jemal A. (2015). Global cancer statistics, 2012. CA Cancer J. Clin. 65, 87–108. 10.3322/caac.21262 25651787

[B119] Tsujimoto Y. Shimizu S. (2005). Another way to die: autophagic programmed cell death. Cell Death Differ. 12(Suppl. 2), 1528–1534. 10.1038/sj.cdd.4401777 16247500

[B120] Tuñón M. J. San-Miguel B. Crespo I. Laliena A. Vallejo D. Álvarez M.. (2013). Melatonin treatment reduces endoplasmic reticulum stress and modulates the unfolded protein response in rabbits with lethal fulminant hepatitis of viral origin. J. Pineal Res. 55, 221–228. 10.1111/jpi.12063 23679826

[B121] Ulivi P. Arienti C. Amadori D. Fabbri F. Carloni S. Tesei A.. (2009). Role of RAF/MEK/ERK pathway, p-STAT-3 and Mcl-1 in sorafenib activity in human pancreatic cancer cell lines. J. Cell. Physiol. 220, 214–221. 10.1002/jcp.21753 19288493

[B122] Ullen A. Farnebo M. Thyrell L. Mahmoudi S. Kharaziha P. Lennartsson L.. (2010). Sorafenib induces apoptosis and autophagy in prostate cancer cells *in vitro*. Int. J. Oncol. 37, 15–20. 10.3892/ijo_00000648 20514392

[B123] Vallejo D. Crespo I. San-Miguel B. Alvarez M. Prieto J. Tuñón M. J.. (2014). Autophagic response in the Rabbit Hemorrhagic Disease, an animal model of virally-induced fulminant hepatic failure. Vet. Res. 45:15. 10.1186/1297-9716-45-15 24490870 PMC3922607

[B124] van der Veen A. G. Ploegh H. L. (2012). Ubiquitin-like proteins. Annu. Rev. Biochem. 81, 323–357. 10.1146/annurev-biochem-093010-153308 22404627

[B125] Verfaillie T. Salazar M. Velasco G. Agostinis P. (2010). Linking ER stress to autophagy: potential implications for cancer therapy. Int. J. Cell Biol. 2010:930509. 10.1155/2010/930509 20145727 PMC2817393

[B126] Walker T. Mitchell C. Park M. A. Yacoub A. Graf M. Rahmani M.. (2009). Sorafenib and vorinostat kill colon cancer cells by CD95-dependent and -independent mechanisms. Mol. Pharmacol. 76, 342–355. 10.1124/mol.109.056523 19483104 PMC2713126

[B127] Wecksler A. T. Hwang S. H. Wettersten H. I. Gilda J. E. Patton A. Leon L. J.. (2014). Novel sorafenib-based structural analogues: *in-vitro* anticancer evaluation of t-MTUCB and t-AUCMB. Anticancer. Drugs 25, 433–446. 10.1097/CAD.0000000000000079 24525589 PMC3947856

[B128] Wellbrock C. Karasarides M. Marais R. (2004). The RAF proteins take centre stage. Nat. Rev. Mol. Cell Biol. 5, 875–885. 10.1038/nrm1498 15520807

[B129] Wilhelm S. M. Adnane L. Newell P. Villanueva A. Llovet J. M. Lynch M. (2008). Preclinical overview of sorafenib, a multikinase inhibitor that targets both Raf and VEGF and PDGF receptor tyrosine kinase signaling. Mol. Cancer Ther. 7, 3129–3140. 10.1158/1535-7163.MCT-08-0013 18852116 PMC12261297

[B130] Wilhelm S. M. Carter C. Tang L. Wilkie D. McNabola A. Rong H.. (2004). BAY 43-9006 exhibits broad spectrum oral antitumor activity and targets the RAF/MEK/ERK pathway and receptor tyrosine kinases involved in tumor progression and angiogenesis. Cancer Res. 64, 7099–7109. 10.1158/0008-5472.CAN-04-1443 15466206

[B131] Yamamoto Y. De Velasco M. A. Kura Y. Nozawa M. Hatanaka Y. Oki T.. (2015). Evaluation of *in vivo* responses of sorafenib therapy in a preclinical mouse model of PTEN-deficient of prostate cancer. J. Transl. Med. 13, 150. 10.1186/s12967-015-0509-x 25953027 PMC4438623

[B132] Yang F. Teves S. S. Kemp C. J. Henikoff S. (2014). Doxorubicin, DNA torsion, and chromatin dynamics. Biochim. Biophys. Acta 1845, 84–89. 10.1016/j.bbcan.2013.12.002 24361676 PMC3927826

[B133] Yang Z. Ming X. F. (2012). mTOR signalling: the molecular interface connecting metabolic stress, aging and cardiovascular diseases. Obes. Rev. 13(Suppl. 2), 58–68. 10.1111/j.1467-789X.2012.01038.x 23107260

[B134] Yi P. Higa A. Taouji S. Bexiga M. G. Marza E. Arma D.. (2012). Sorafenib-mediated targeting of the AAA^+^ ATPase p97/VCP leads to disruption of the secretory pathway, endoplasmic reticulum stress, and hepatocellular cancer cell death. Mol. Cancer Ther. 11, 2610–2620. 10.1158/1535-7163.MCT-12-0516 23041544

[B135] Yin J. Q. Wan Y. (2002). RNA-mediated gene regulation system: now and the future (Review). Int. J. Mol. Med. 10, 355–365. 10.3892/ijmm.10.4.355 12239579

[B136] Yoneda T. Imaizumi K. Oono K. Yui D. Gomi F. Katayama T.. (2001). Activation of caspase-12, an endoplastic reticulum (ER) resident caspase, through tumor necrosis factor receptor-associated factor 2-dependent mechanism in response to the ER stress. J. Biol. Chem. 276, 13935–13940. 10.1074/jbc.M010677200 11278723

[B137] Yu C. Bruzek L. M. Meng X. W. Gores G. J. Carter C. A. Kaufmann S. H.. (2005). The role of Mcl-1 downregulation in the proapoptotic activity of the multikinase inhibitor BAY 43-9006. Oncogene 24, 6861–6869. 10.1038/sj.onc.1208841 16007148

[B138] Yuan H. Li A.-J. Ma S.-L. Cui L.-J. Wu B. Yin L.. (2014). Inhibition of autophagy significantly enhances combination therapy with sorafenib and HDAC inhibitors for human hepatoma cells. World J. Gastroenterol. 20, 4953–4962. 10.3748/wjg.v20.i17.4953 24833845 PMC4009527

[B139] Yuan H. X. Russell R. C. Guan K. L. (2013). Regulation of PIK3C3/VPS34 complexes by MTOR in nutrient stress-induced autophagy. Autophagy 9, 1983–1995. 10.4161/auto.26058 24013218 PMC4028342

[B140] Zhai B. Hu F. Jiang X. Xu J. Zhao D. Liu B.. (2014). Inhibition of Akt reverses the acquired resistance to sorafenib by switching protective autophagy to autophagic cell death in hepatocellular carcinoma. Mol. Cancer Ther. 13, 1589–1598. 10.1158/1535-7163.MCT-13-1043 24705351

[B141] Zhai B. Jiang X. He C. Zhao D. Ma L. Xu L.. (2015). Arsenic trioxide potentiates the anti-cancer activities of sorafenib against hepatocellular carcinoma by inhibiting Akt activation. Tumour Biol. 36, 2323–2334. 10.1007/s13277-014-2839-3 25416439

[B142] Zhang C. Z. Wang X. D. Wang H. W. Cai Y. Chao L. Q. (2015). Sorafenib inhibits liver cancer growth by decreasing mTOR, AKT, and PI3K expression. J. BUON 20, 218–222. 25778319

[B143] Zheng B. Zhu H. Gu D. Pan X. Qian L. Xue B.. (2015). MiRNA-30α-mediated autophagy inhibition sensitizes renal cell carcinoma cells to sorafenib. Biochem. Biophys. Res. Commun. 459, 234–239. 10.1016/j.bbrc.2015.02.084 25712526

[B144] Zhou F. Yang Y. Xing D. (2011). Bcl-2 and Bcl-xL play important roles in the crosstalk between autophagy and apoptosis. FEBS J. 278, 403–413. 10.1111/j.1742-4658.2010.07965.x 21182587

